# ^19^F NMR as a tool in chemical biology

**DOI:** 10.3762/bjoc.17.28

**Published:** 2021-01-28

**Authors:** Diana Gimenez, Aoife Phelan, Cormac D Murphy, Steven L Cobb

**Affiliations:** 1 Department of Chemistry, Durham University, South Road, Durham, DH13LE, UK; 2 UCD School of Biomolecular and Biomedical Science, University College Dublin, Belfield, Dublin 4, Ireland

**Keywords:** biotransformation, chemical biology, fluorine, ^19^F NMR, probes, protein structure

## Abstract

We previously reviewed the use of ^19^F NMR in the broad field of chemical biology [Cobb, S. L.; Murphy, C. D. *J. Fluorine Chem.* **2009,** *130,* 132–140] and present here a summary of the literature from the last decade that has the technique as the central method of analysis. The topics covered include the synthesis of new fluorinated probes and their incorporation into macromolecules, the application of ^19^F NMR to monitor protein–protein interactions, protein–ligand interactions, physiologically relevant ions and in the structural analysis of proteins and nucleic acids. The continued relevance of the technique to investigate biosynthesis and biodegradation of fluorinated organic compounds is also described.

## Introduction

Although fluorine is abundant in the environment, it is not a nutrient nor is it a feature of biochemistry for most species [1]. This reflects its low bioavailability, as it is typically found in insoluble minerals, and its physicochemical properties, in particular its high redox potential and the poor reactivity of the fluoride ion in aqueous solution. Despite its near-absence in biology, it is a particularly important element in the broader space of chemical biology [2]. Many commercial and industrial compounds are fluorinated, including refrigerants, degreasers, drugs, pesticides and anti-stick materials, and consequently there is a high degree of interaction between fluorinated compounds and nature [3]. Furthermore, owing to the lack of naturally-occurring fluorinated compounds, fluorine is a useful probe to investigate structure and mechanism of biological molecules. Central to these studies is fluorine-19 nuclear magnetic resonance spectroscopy (^19^F NMR), which allows the user to readily visualize changes in chemical shift and splitting pattern depending on the changes of the (biological) environment, without substantial purification or processing of samples. In 2009 we published a review describing the application of ^19^F NMR in chemical biology [4] and here we present the advances that have been made in this field since then.

## Review

### Structural analysis of macromolecules using ^19^F NMR

In order to elucidate their various functions, it is essential to have a detailed understanding of both the structure of biological molecules and the way in which they interact in their environment and with one another. With regards to probing both the structure and the interactions between biomolecules in complex settings the analytical tool ^19^F NMR has become invaluable. Some key highlights of how ^19^F NMR has been employed in this area are given in the following section.

#### Recent advances in protein ^19^F labelling

^19^F NMR offers an attractive option for investigating the interactions between proteins and other biomolecules such as nucleic acids. Many of the advantages of ^19^F NMR have already been discussed but it is worth highlighting that it is a particularly useful technique to study large proteins that cannot easily be probed by conventional NMR experiments. Given that fluorine atoms (e.g., ^19^F labels) are not naturally present in proteins, a key element to establishing ^19^F NMR in this area has been the development of methods that can be used to give access to ^19^F-labelled proteins.

**Figure 1:**
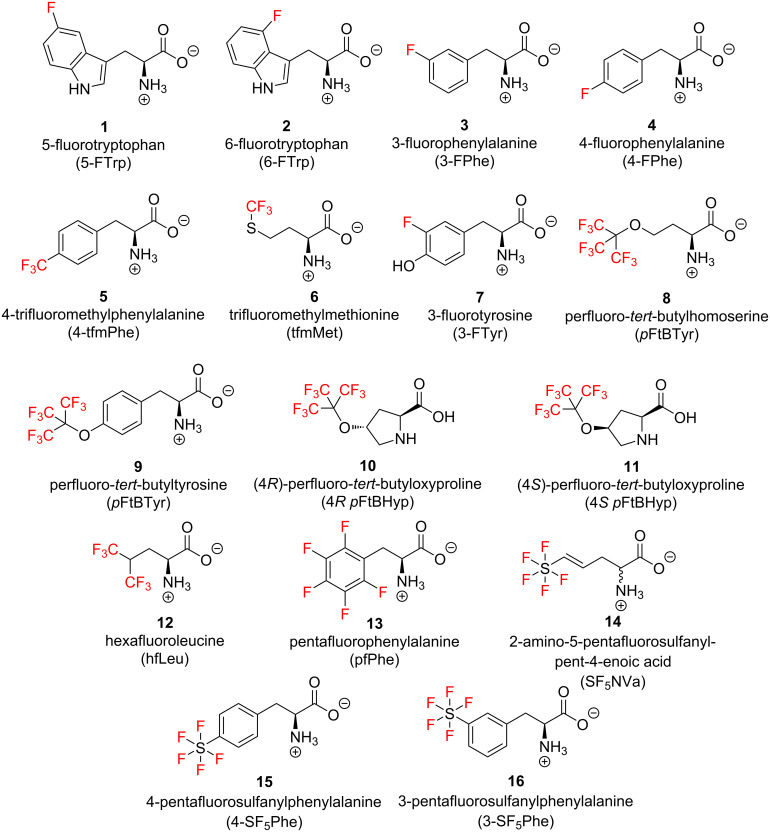
Selected examples of ^19^F-labelled amino acid analogues used as probes in chemical biology.

Methods for the introduction of unnatural amino acids into proteins have been reviewed extensively elsewhere [5–7] and here we will only discuss the most recent advances. The chemical structures of a selection of ^19^F-labelled amino acid analogues that have been utilized in ^19^F NMR studies in chemical biology are shown in Figure 1. In general, relatively small monofluorinated amino acids such as **1**–**4**, can be biosynthetically incorporated directly into proteins by including the fluorinated amino acid in the growth medium of a suitable auxotrophic bacterium [8]. However, a challenge when producing ^19^F-labelled proteins using this technique is that the natural amino acid translation process incorporates the specific fluorinated analogue in all the locations where the original amino acid was present, resulting in a “global” labelling of the protein. This can result in a variety of unwanted side effects, including, in some cases, structure disruption and possible ^19^F NMR spectral overcrowding [9]. To achieve controlled site-specific amino acid incorporation the methodology pioneered by Schultz et al. [10] is often employed. Here orthogonal amber suppressor tRNA with a paired tRNA synthetase enables the introduction of a broader variety of ^19^F-labelled acids at single site-specific positions. However, while these systems can be highly precise for certain proteins, the expression yields are strongly context dependent and can lead to poor levels of fluorinated amino acid incorporation [11–12]. To overcome this issue, Otting and co-workers have recently developed a synthetic strategy, based on a continuous exchange cell-free system (CECF), in which a key chitin-binding mutant of release factor RF1 can be removed under conditions that maintain the full activity of the S30 extract, thereby minimizing the incidence of premature translation termination and improving the expression yields by suppressing the production of truncated proteins [13]. This strategy has proven efficient for the site-specific incorporation of up to four trifluoromethylphenylalanines (**5**, Figure 1) within West Nile virus NS2B‐NS3 protease (WNVpro) and for the incorporation of 5-fluorotryptophan (**1**) and 6-fluorotryptophan (**2**) in streptavidin [14].

**Figure 2:**
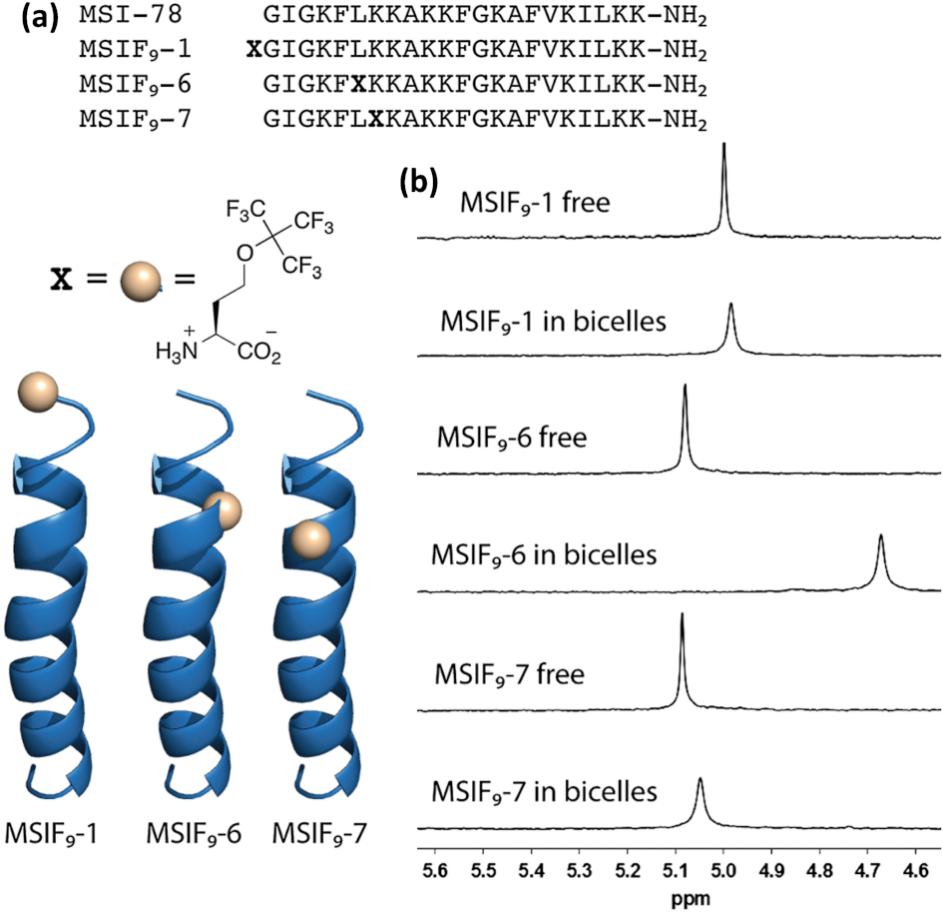
(a) Sequences of the antimicrobial peptide MSI-78 and *p*FtBSer-containing analogs and cartoon representations illustrating the helical location of *p*FtBSer residues. (b) ^19^F NMR spectra showing the changes associated with MSIF9-1, MSIF9-6 and MSIF9-7 upon binding to bicelles. Figure 2 is adapted from [15]. Copyright © 2013 European Peptide Society and John Wiley & Sons, Ltd. Used with permission from Benjamin C. Buer et al., “Perfluoro‐*tert*‐butyl‐homoserine as a sensitive ^19^F NMR reporter for peptide–membrane interactions in solution”, Journal of Peptide Science, John Wiley and Sons.

Parallel to the development of these new strategies for recombinant fluorinated protein production, significant efforts have been made within the last decade to expand the pool of available amino acids that can be used as ^19^F NMR reporters. Aromatic amino acids have been preferentially used as ^19^F NMR probes both in binding and in conformational studies owing to their ready availability. However, more recently fluoro-aliphatic, and in particular perfluoro-aliphatic amino acids, have become increasingly prominent in ^19^F NMR studies owing to their improved spectral properties. Among the first perfluorinated amino acids to be reported was perfluoro-*tert*-butylhomoserine (**8**), which was synthesized by Marsh and co-workers and incorporated into a variety of antimicrobial peptides, including MSI-78, for their NMR study on lipid bicelles (Figure 2) [15]. In contrast to more traditional amino acids, such as **1**–**4**, the perfluorinated *tert*-butyl moiety in **8** has nine equivalent fluorine atoms and no coupled hydrogens, so it gives rise to a characteristic high intensity ^19^F NMR singlet. As shown by the authors, all peptides where **8** was introduced could be detected even at concentrations as low as 5 μM, demonstrating that *p*FtBSer is a highly sensitive tool to study binding events by way of ^19^F NMR chemical shift and nuclear relaxation changes.

In addition to homoserine, perfluorinated analogues of both tyrosine [16] (*p*FtBTyr, **9**) and proline [17] (4*R* and 4*S*-*p*FtBHyp, **10** and **11**, Figure 1) have also been utilised as ^19^F NMR probes, both showing promise as valuable tools for biomolecular studies. Particularly impressive is that by incorporating *p*FtBTyr (**9**) into a model peptide, Tressler and Zondlo were able to detect the presence of a sharp singlet in the ^19^F NMR spectrum even at nanomolar peptide concentrations. It is worth noting here that, as recently reviewed in detail by Koksch [18–19], while some perfluorinated amino acids such as hexafluoroleucine (**12**, Figure 1) and pentafluorophenylalanine (**13**, Figure 1) might exhibit, in general, low α-helix propensities, others such as **8** have been found to promote significant levels of α-helix formation [15]. This specific conformational propensity seen in **8** allowed, for example, for its non-disruptive incorporation into the α-helical motif of several estrogen receptor (ER) co-activator peptides and enabled the sensitive detection of their protein–peptide interaction inhibition by the ER antagonist tamoxifen [20]. Significantly, different secondary structure conformational preferences were also found among the diastereomers of perfluoro-*tert*-butyloxyproline, with the 4*R* analogue exhibiting a higher propensity for the polyproline helix structure than the 4*S* [17]. This differentiated conformational bias observed highlights the potential application of these amino acids as unique probes for molecular recognition studies.

The synthesis of unnatural amino acids carrying novel fluorine-based functionalities such as -SF_5_ has also recently achieved considerable interest. For instance, the work carried out within the Welch group, who reported the synthesis and NMR conformational characterization of the first heptapeptide containing a pentafluorosulfanylated aliphatic amino acid, (*E*)-2-amino-5-(pentafluorosulfanyl)pent-4-enoic acid (**14**, Figure 1), SF_5_NVa [21]. Most recently, Cobb et al. [22] reported the synthesis of several pentafluorosulfanyl phenylalanine derivatives with suitable protecting groups to allow incorporation into peptides through common solid-phase peptide synthesis (SPPS) methods (**15** and **16**, Figure 1).

**Figure 3:**
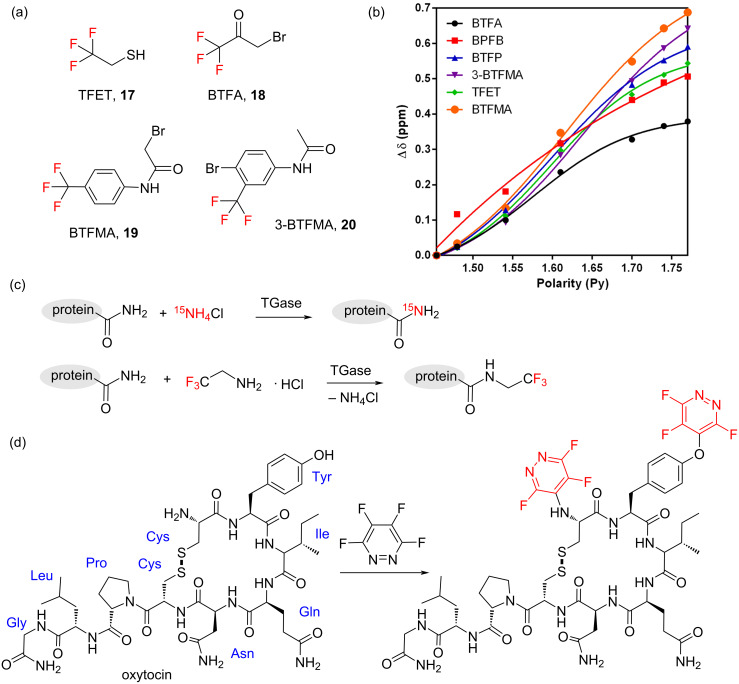
(a) Chemical structures of a selection of trifluoromethyl tags. (b) Comparative analysis showing the changes in ^19^F chemical shift (Δδ) of various CF_3_ tags as a function of solvent polarity. (c) Schematics showing the enzymatic isotope labelling of glutamine γ-carboxamide groups using transglutaminase (TGase) and ^15^N-ammnonium chloride (^15^NH_4_Cl) or ^19^F-2,2,2-trifluoroethylamine hydrochloride (TFEA-HCl). (d) Example of a cyclic peptide hormone, oxytocin, and its perfluoropyridazine “tagged” analogue. Figure 3b was adapted from [28]; Figure 3d was reprinted from [30].

A second well-established methodology for the ^19^F isotopic labelling of protein and peptides involves the post-translational chemical conjugation of an ^19^F probe to specific amino acids present within the protein, typically cysteine and lysine residues. This approach is attractive as it can be directly applied to isotopically enrich unlabelled proteins, which may have been obtained previously from natural sources or as recombinant proteins under less demanding cell growth conditions. As shown in Figure 3, these fluorine tags consist, in general, of reactive trifluoromethyl derivatives such as 2,2,2-trifluoroethanethiol (TFET, **17**) [23], 3-bromo-1,1,1-trifluoroacetone (BTFA, **18**) [24] and fluorinated haloacetamides [25] that can react with nucleophilic side chains on the protein of interest. This approach has been extensively applied to study both soluble proteins [26] and membrane proteins [27]. Fluorine tags such at TFET (**17**) or BFTA (**18**) offer a good degree of sensitive and narrow ^19^F NMR line widths due to rapid rotation about the trifluoromethyl symmetry axis. Thus, they are very effective tools when studying small conformational changes in proteins and other real-time events, even if relatively low concentrations are employed. In addition, these fluorine tags have proven to be sensitive to very subtle changes in their environment, providing further local-site specific conformational information [28]. Recent advances in this area include the tags based on fluorinated phenylacetamides, such as the 2-bromo-*N*-(4-(trifluoromethyl)phenyl)acetamide (BTFMA, **19**) and *N*-(4-bromo-3-(trifluoromethyl)phenyl)acetamide (3-BTFMA, **20**) developed by Manglik et al. to study the structural dynamics of the cytoplasmic domain of the β2-adrenergic receptor (β2AR) [29]. In this work conjugation of the CF_3_ group to an aromatic ring was shown to give rise to substantially improved ^19^F NMR chemical shift sensitivities over the more traditional thiol-specific trifluoromethyl tags (Figure 3b) [28].

In addition to chemical modification, novel methodologies are being investigated that enable the enzymatic post-translational modification of non-nucleophilic residues, such as glutamate [31]. As recently demonstrated by Kojima and co-workers, recombinant protein transglutaminase (TGase) could be used to catalyse the chemical replacement of the γ-carboxyamide groups in Glu residues by free 2,2,2-trifluoroethylamine (TFEA-HCl) in model binding protein FKB12. This resulted in the site-specific incorporation of CF_3_CH_2_- motifs into these amino acids (Figure 3c). This ^19^F-labelling strategy clearly offers new opportunities in the area as it can be easily combined with the aforementioned chemical approaches based on cysteine and lysine modification.

It is worth noting that for small proteins and peptides the chemical incorporation of the desired fluorinated amino acids using SPPS protocols still remains the method of choice, as it enables the site-specific introduction of the ^19^F NMR probe. In addition, SPPS offers a powerful technique to access uniquely labelled peptides and proteins when combined with new strategies for peptide stapling and tagging using perfluoroaromatic reagents. Perfluorophenyl, perfluoropyridyl and perfluoropyridazinyl labelled peptides can now all be accessed, expanding even further the tool-box of ^19^F NMR probes available for protein studies (Figure 3d) [30,32].

#### Protein-observed binding interactions

^19^F NMR is a valuable analytical tool to study the binding interactions between a variety of biological substrates and ^19^F-labelled proteins. It has also become a widely adopted method for ligand screening, enabling the evaluation of small molecule–protein interactions over a wide range of affinities. In protein-observed fluorine NMR experiments (PrOF) binding events can be readily monitored using simple 1D techniques, and the dissociation binding constant of ligands (*K*_d_) determined from the changes in the chemical shift of the labelled nuclei within the protein. Intuitively, the largest chemical shift perturbations are expected to be seen for residues in the closest proximity to the bound ligand. An additional advantage of this method is that signal acquisition in these experiments is typically rapid, as large signal-to-noise ratios are not needed to obtain precise indication of the positive presence of binding interactions [33].

**Figure 4:**
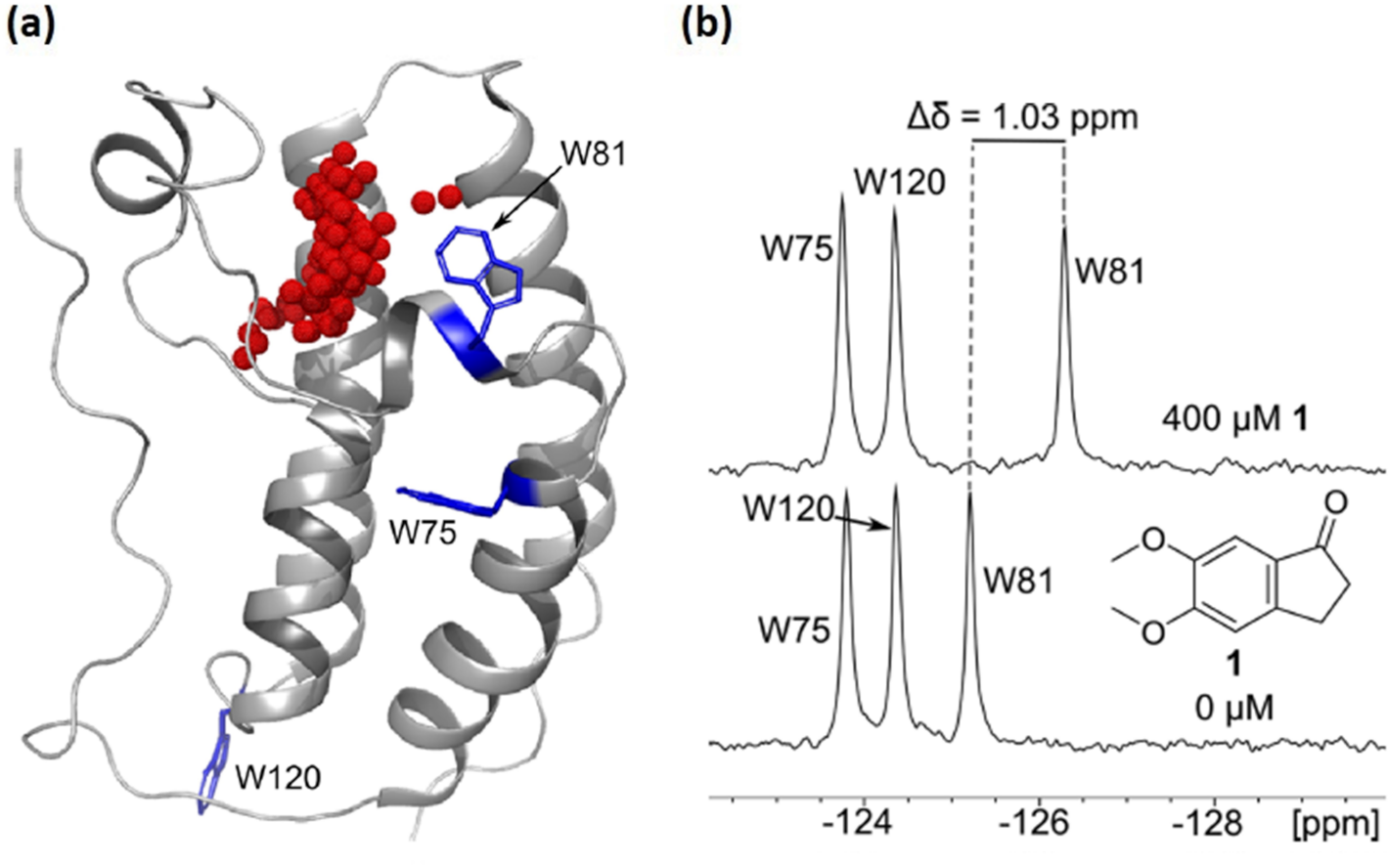
(a) First bromodomain of Brd4 with all three tryptophan residues displayed in blue and labelled by residue number. Red spheres indicate the acetylated lysine binding site (generated by SiteMap). PDB ID: 3UVW. (b) PrOF NMR spectrum of 5-FTrp-Brd4 and a selected ligand. In the presence of the ligand, the resonance for W81 is significantly shifted upfield consistent with binding near the acetylated lysine interaction site of Brd4. Adapted with permission from [34]. © 2016 American Chemical Society.

Pomerantz and co-workers analysed the difference in NMR chemical shift perturbations for the ^1^H-(NH) and ^19^F nuclei in native tryptophan, and 5-FTrp-labelled bromodomain protein Brd4 against a panel of ligands (Figure 4) [34]. ^1^H NMR was found to be 6–20 times less responsive than fluorine with regards to chemical shift perturbations. Indeed, the high resolution observed in the fluorine spectra enabled the direct calculation of most of the ligand binding constants. The superior capacity of ^19^F NMR to monitor binding events was also noted by Richards et al., when studying the interaction of human protein disulphide isomerase (hPDI) to Δ-somastatin [35]. In this work there was improved precision in the analysis of the dissociation constants due to the higher spectrum resolution and greater chemical environment sensitivity of the ^19^F nuclei when compared to that offered by the ^15^N.

Due to the fact that different regions of a protein give rise to unique NMR resonances, protein-observed experiments using multi-site labelled proteins can be used to provide information on changes in the chemical environment of the protein surface, which can be used to characterize binding-sites and/or conformational changes upon binding. For example, ^19^F NMR studies on cysteine-labelled ß2-adrenergic receptor (β2AR) using either a ^19^F-BTFA [29] or a ^19^F-TFET [36] tag were used to explore the differential long range conformational effects induced by agonists, inverse agonists, and partial agonists on GPCR signalling. As exemplified by Liu et al. [36], in the case of multiply site-specific labelled ^19^F-TFET-β2AR, changes in the NMR signals monitored within different helices of the protein revealed that agonist binding primarily shifted the equilibrium towards the G protein-specific active state of helix VI. In contrast, β-arrestin-biased ligands predominantly affected the conformation of specifically helix VII, providing thus insights into the structural plasticity of β2AR receptor.

By taking a novel approach to the use of PrOF NMR, Stadmiller et al. have recently developed a pioneering methodology based on 1D ^19^F NMR line shape analysis that can be utilized to simultaneously determine both thermodynamic equilibrium binding constants (*K*) and kinetic rates of association (*k*_on_) and dissociation (*k*_off_) in protein–ligand binding events [37]. In order to achieve this, they analysed the binding of four different proline-rich peptides to a 5-FTrp-labelled Src homology 3 (SH3) recognition protein domain. By comparing the results obtained using both, the newly proposed ^19^F NMR-based analysis and the traditional 2D ^15^N,^1^H-HSQC experiments they could demonstrate that simple ^19^F NMR line shape analysis achieved comparable high quantitative accuracy in the determination of all binding parameters. In addition, qualitative residue-specific information was also provided, but with a much improved and straightforward post-acquisition analysis procedure. It is clear that this more user-friendly methodology has the potential to have a significant impact in future studies of protein–ligand interactions, protein folding, and potentially small-molecule library screening, as it can broaden the accessibility of quantitative NMR spectroscopy to a wider range of laboratories.

#### Ligand-observed protein binding interactions

In addition to protein-observed fluorine (PrOF) NMR spectroscopy, ligand-observed fluorine NMR spectroscopy for drug screening has also evolved in the last decade to become a key tool to study the binding of drug candidates to target proteins. ^19^F NMR-based screening, which was developed by Dalvit et al., includes fluorine chemical shift anisotropy and exchange for screening (FAXS) [38] which is a binding assay, and fluorine atoms for biochemical screening (*n*-FABS) methodology which is a functional assay [39–40]. Both of these methods have been successfully used for ligand-based screening, fragment-based functional screening and dynamic library screening, and have been recently reviewed in detail by Dalvit and Vulpetti [41–42]. The main advantages of these methods for lead compound screening is that they offer a platform not only for the rapid high-throughput screening of multiple protein small ligands, but also for the direct screening of functional inhibitors of much larger and complex biomolecules, such as enzymes.

**Figure 5:**
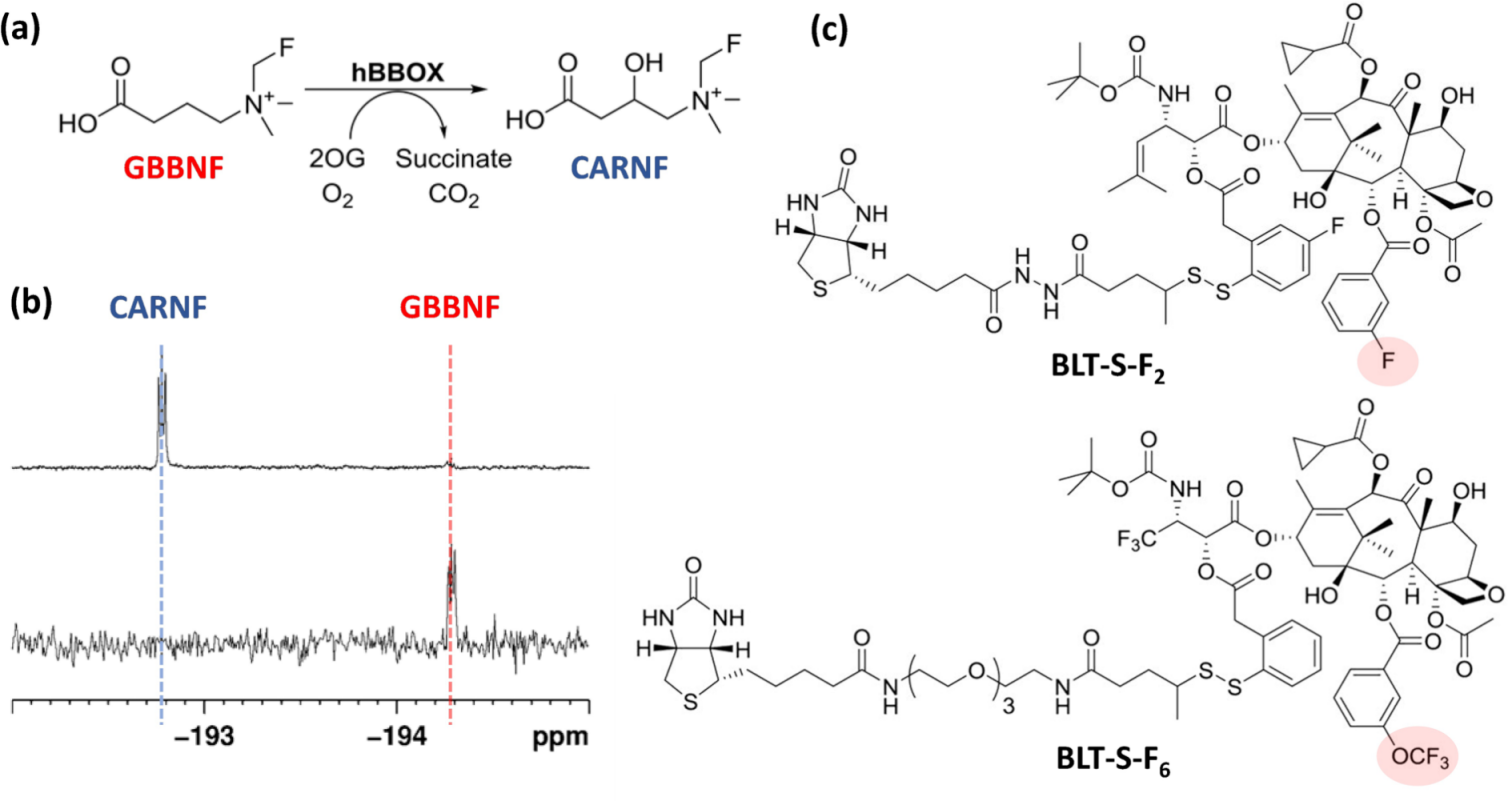
(a) Enzymatic hydroxylation of GBBNF in the presence of hBBOX (b) ^19^F NMR spectra showing the conversion of GBBNF to CARNF by psBBOX. (c) Chemical structure of BLT-F2 and BLT-S-F6. Figure 5a and Figure 5b were reproduced and adapted from [43], Figure 5c was reproduced from [44].

In a recent study exemplifying this application, ^19^F NMR has been employed to investigate the metabolism of carnitine. In animals, carnitine is biosynthesised from trimethyllysine in four enzyme-catalysed steps, which involves in the last step the action of the γ-butyrobetaine hydroxylase enzyme (hBBOX) (Figure 5a–c). As the fluorine shift of the metabolised product is distinctively different from the shift of the precursor, by producing a novel fluoromethyl analogue of the γ-butyrobetaine substrate (GBBNF) Rydzik et al. were able to monitor carnitine biosynthesis through hBBOX-catalysed GBBNF hydroxylation, both in vitro and in cell lysates [43]. Moreover, by using a competitive substrate for the enzyme, inhibition experiments could be directly employed to determine the IC_50_ values in the basis of fluoride release, and the extent of GBBNF turnover investigated in the presence of cell extracts.

Ojima and collaborators have also employed functional ligand-observed NMR experiments to develop a range of novel 3′-difluorovinyltaxoids, such as BLT-F2 and BLT-S-F6 (Figure 5c), that are active against drug-sensitive and multidrug-resistant (MDR) human cancer cell lines [44]. Here, the incorporation of CF_3_ and CF_3_O groups as NMR reporters into the tumour-targeting drug conjugates enabled the direct investigation of the mechanism of the pro-drug metabolic cleavage and the pharmacophore release by real-time ^19^F NMR analysis. ^19^F NMR was also employed as a convenient technique to study a range of factors that might influence the plasma and metabolic stability of the lead compounds, proving itself as a valuable alternative to more challenging conventional HPLC or ^1^H NMR analyses.

**Figure 6:**
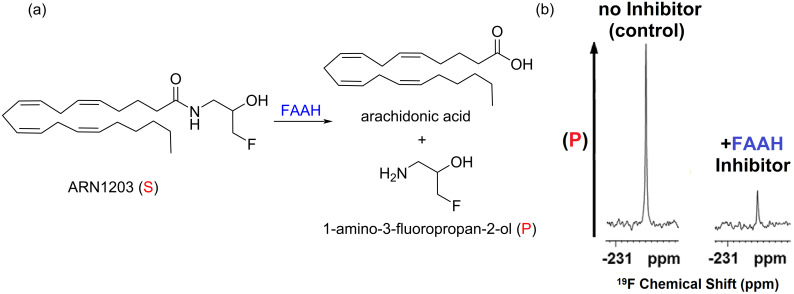
(a) In-cell enzymatic hydrolysis of the fluorinated anandamide analogue ARN1203 catalyzed by hFAAH. Only the fluorinated substrate and the 1-amino-3-fluoropropan-2-ol product are detected in the ^19^F NMR spectra. (b) ^19^F NMR spectra of ARN1203 incubated for 2.5 h in hFAAHeHEK293 intact cells in the presence and absence of an hFAAH activity inhibitor. Figure 6b was reproduced and adapted from [45].

It is also worth noting that recent investigations within the Dalvit group have further expanded the applications of *n*-FABS into the screening and direct IC_50_ measurement of bioactive substrates within intact living cells [45]. By studying mammalian cells expressing the membrane protein fatty acid amide hydrolase (FAAH), they showed that it is possible to screen and identify target inhibitors in situ using ^19^F NMR spectroscopy (Figure 6). For this, the activity of the membrane-bound FAAH enzyme was evaluated by monitoring the hydrolysis of a fluorinated anandamide analogue ARN1203, a previously reported FAAH substrate, to arachidonic acid and 1-amino-3-fluoropropanol in the presence and absence of a broad range of known FAAH inhibitors with widely different potencies. The results clearly showed that the proposed *n*-FABS method was successful in detecting strong inhibitors of FAAH activity in cells. Also, it allowed the accurate quantification of the corresponding IC_50_ values, all of which were consistent with those obtained in previous work but measured in cell membrane extracts [46].

### Structural analysis of macromolecules using ^19^F NMR

The three-dimensional (3D) structure of biomacromolecules and their conformational dynamics are often the starting point for the development of an in-depth understanding of their function and mode of action. Owing to the large size of biomacromolecules, the high complexity of the processes in which they participate and the natural environment in which they occur, an integrated analytical approach is often required to succeed in revealing their functional architecture. Such an approach typically includes the use of a variety of high-resolution proteomic tools, cryo-electron microscopy and X-ray crystallography to achieve full molecular characterization at the atomic level. However, macromolecules such as DNA/RNA and proteins are not static entities, for which also complementary dynamic and targeted local site-specific conformational information is needed to fulfil our understanding on how these assemblies perform their biological role and function in vivo. It is in this regard that ^19^F NMR has proven to be a valuable analytical tool, providing simultaneous access to both structural and dynamic information that would be difficult to obtain using traditional ^13^C, ^1^H and ^15^N,^1^H-HSQC NMR techniques.

#### Protein folding

From the late 1960s and early 1970s ^19^F NMR has firmly established itself as a highly versatile and powerful analytical tool for studying both protein structure and conformational changes within proteins [47]. For additional information on the development of the field of ^19^F NMR protein studies readers are encouraged to consult the excellent earlier reviews written by Gerig [48] and Danielson and Falke [49].

However, over the last 20 years the application of ^19^F NMR in this area has shifted from the mere study of proteins and peptides as static entities, to the detailed analysis of the much more sophisticated protein transient conformational states that are produced upon substrate interaction, folding or binding. Thus, it is possible to accurately track the complete chain of events that take place within the molecule upon interaction and/or folding. Given that the intermediates involved in protein folding and binding are difficult to observe owing to the short-lived nature of the states and their marginal concentration, high sensitivity ^19^F NMR has been employed to offer unique opportunities for their sampling and characterization in situ [50].

**Figure 7:**
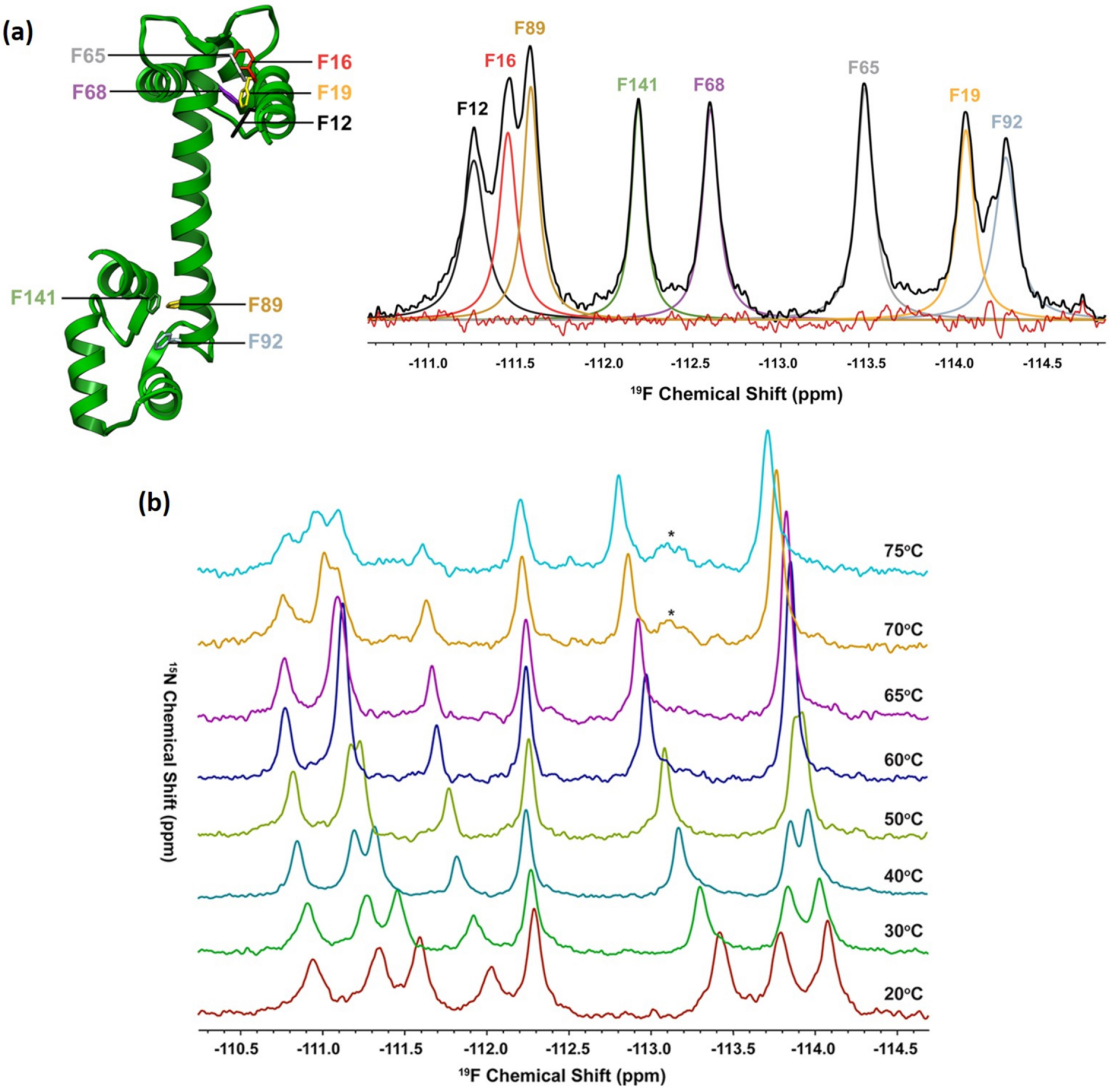
(a) X-ray crystal structure of CAM highlighting the location the phenylalanine residues replaced by 3-FPhe and the corresponding deconvoluted ^19^F NMR spectrum of the fractionally labelled peptide (PDB file 3CLN). (b) ^19^F NMR spectra of 70% 3-FPhe fractionally labelled CAM as a function of temperature. Adapted with permission from [51]. © 2013 American Chemical Society.

Prosser et al. demonstrated the utility of this approach when employing a combination of variable temperature (VT) ^19^F NMR analysis and CD spectroscopy to study the near-native thermal folding intermediate structure of calmodulin (CAM) (Figure 7) [51]. To this end, CAM was biosynthetically enriched with 3-fluorophenylalanine (3-FPhe) and the ^19^F NMR signals corresponding to each one of the eight 3-FPhe residues present resolved and assigned in the spectra for the native state. Changes in the ^19^F NMR signals produced upon increasing the temperature were then used to map the evolution of the protein along the heat-induced denaturalization curve and to confirm the presence of an intermediate transient structure at 67 °C. Notably, single Lorentzian lines were observed for all 3F-Phe labelled sites regardless of the temperature employed, signifying either a single state at low temperatures or fast exchange between the native and near-native states at higher temperatures (40–70 °C). The NMR data obtained also provided evidence of the rapid ring flipping of the 3-FPhe probes, inherent to the side-chain dynamics within the protein hydrophobic interior [52–54]. ^19^F NMR has also been used to characterize in more detail the topography of the CAM conformational changes by using molecular contrast agents. ^19^F NMR solvent-induced isotope shift experiments, resulting from replacement of H₂O with D₂O, provide a robust measure of the relative degree that a specific labelled position is exposed to the solvent [55]. Complementary to this technique, the use of dissolved O_2_ as a paramagnetic agent, having a relatively short electronic relaxation time, gives rise to paramagnetic shifts and spin–lattice relaxation rate enhancements that can be directly correlated with its accessibility to the ^19^F-spin of interest [55–56]. This way, the combined ratio derived from both experiments was employed as a convenient measure of the solvent exposure and local hydrophobicity changes experienced at each individual fluorinated residue upon heating. Ultimately, ^19^F NMR enables profiling of the specific position and nature of the conformational perturbations that the protein undergoes as it progresses from its native state along the heat-denaturation pathway [51,57].

**Figure 8:**
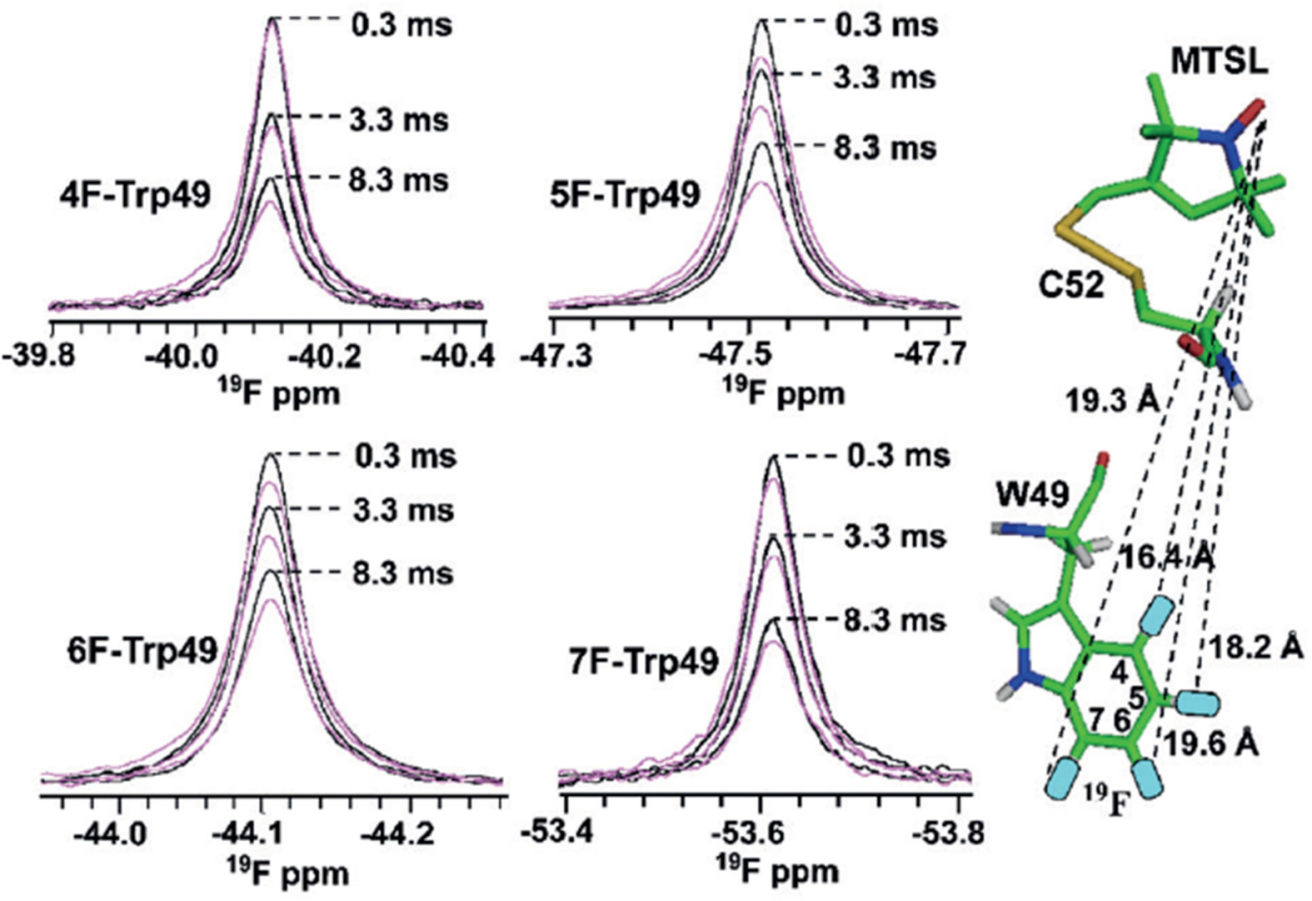
^19^F PREs of 4-F, 5-F, 6-F, 7-FTrp49 containing MTSL-modified ^S52C^CV-N. The ^19^F NMR resonances of oxidized (magenta) versus reduced (black) 4-F, 5-F, 6-F, and 7-FTrp49 ^S52C^CV-N are superimposed for 0.3, 3.3, and 8.3 ms relaxation delays. PRE-derived distances are shown by dashed lines on the model. Figure 8 is reproduced from [58]. © 2016 WILEY‐VCH Verlag GmbH & Co. KGaA, Weinheim. Used with permission from Elena Matei et al., “^19^F Paramagnetic Relaxation Enhancement: A Valuable Tool for Distance Measurements in Proteins”, *Angew. Chem., Int. Ed.*, John Wiley and Sons.

In a much broader context, ^19^F NMR paramagnetic relaxation enhancement experiments (PREs), have been shown to constitute a versatile tool for extracting quantitative structural distance information in selectively ^19^F-labelled proteins. As a proof of concept, Matei et al. [58] demonstrated the applicability of this approach in studying the HIV-inactivating lectin cyanovirin-N protein as a model system (^S52^CCV-N). Single fluorine atoms were introduced at the 4-, 5-, 6- or 7-positions of Trp49 and the 4-position of Phe4, Phe54, and Phe80 (Figure 8). Simultaneously, the paramagnetic nitroxide spin label was chemically attached to the protein by using two available Cys residues at positions 50 or 52. As the presence of an unpaired electron in the tag increases the nuclear relaxation rates of the residues that are in spatial proximity, it resulted in their spectral signal attenuation. Due to this effect being inversely correlated with the distance between the probes, the transverse ^19^F-PRE rate (^19^F-Γ2) was directly employed for the determination of the average distance between the ^19^F nucleus and the paramagnetic centres, within a range of 12–24 Å. Overall, the work showed the potential applicability of ^19^F NMR PREs as excellent alternative parameters for the quantitative analysis of site-specific intramolecular distances, with a more particular significant value in the exploration of large proteins and macromolecular complexes where substrate spectral crowding and/or background incompatibility prevents the use of other nuclei such as ^1^H or ^15^N PREs.

^19^F NMR has also been applied to study the conformational heterogeneity and dynamics of a broad range of proteins and peptides upon their interaction with model lipid vesicles [59], micelles [60] and bicelles [61]. It has enabled the quantification and mechanistic characterization of intermolecular dynamic processes such as protein dimerization, oligomerization and fibrillation, even within large and especially complex systems [62–63].

**Figure 9:**
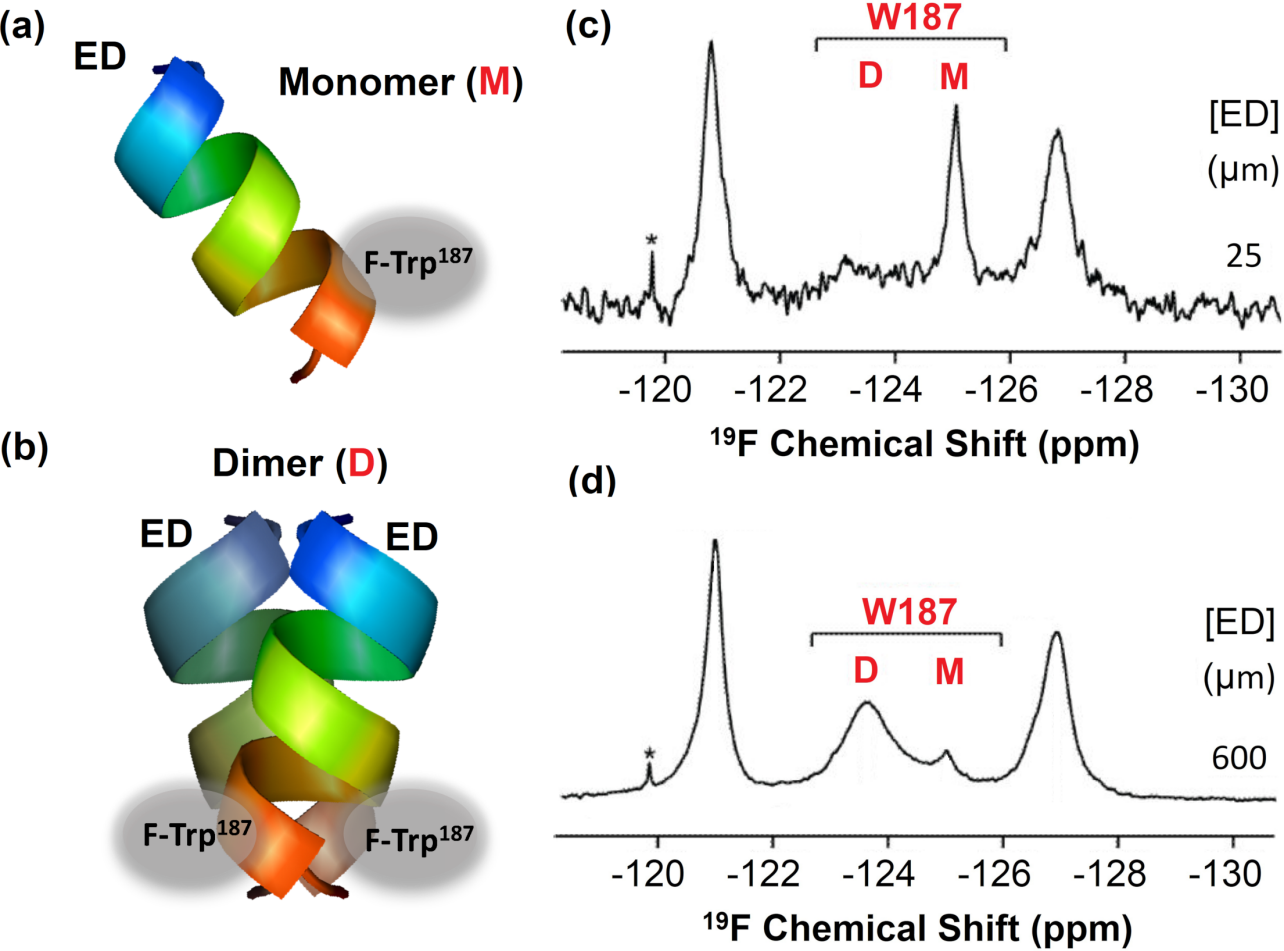
^19^F NMR as a direct probe of Ud NS1A ED homodimerization. Schematic representation showing the location of the 5-F-Trp residue in the monomer (a) and dimer (b) structure of Ud NS1A ED domain. (c, d) Concentration dependence of the ^19^F NMR signal of 5-FTrp187 within Ud NS1A ED in low salt pH 8 buffer. Figure 9 was reproduced and adapted from [64].

In a recent study, Aramini et al. employed ^19^F NMR to investigate the conformational dynamics within the interacting interface of non-structural protein 1 (NS1A) homodimer (Figure 9) [64]. Protein NS1A is a highly conserved virulence factor from influenza virus (H3N2) comprised of an N-terminal double-stranded RNA (dsRNA)-binding domain (RBD) and a multifunctional C-terminal effector domain (ED), each of which can independently form symmetric homodimers. By labelling NS1A with 5-FTrp at the different sites within the ED, Aramini et al. were able to demonstrate that a specific labelled residue, 5-FTrp187, exhibited indeed a higher degree of ^19^F NMR line width broadening when compared to the other fluorinated amino acids incorporated upon interaction. These results demonstrated that the site-specific ^19^F signal of 5-FTrp187 could be directly employed as a reliable reporter to monitor the monomer/dimer exchange dynamics. Moreover, it could also be employed to characterize the ED helix–helix dimer interface in more detail by means of additional 1D ^19^F T1, T2, and CPMG relaxation dispersion experiments and solvent-induced isotope shift effects. This study also highlighted the particular advantages of employing ^19^F NMR fluorine probes strategically labelled in the second ring of the Trp indole moiety to directly measure conformational exchange phenomena at protein-protein interfaces mediated by such residues [65].

Correct folding is essential for normal biological function of proteins [66]. The formation and accumulation of misfolded proteins has been shown to be a common pathological feature in a significant number of human disorders, including neurodegenerative Alzheimer’s and Parkinson’s diseases and several metabolic type II diabetes conditions. So far, the application of real-time NMR to characterize amyloid formation has been very limited [67–68], largely due to the spectral overcrowding of the 1D ^1^H NMR spectra and the difficulties associated with performing multidimensional experiments at a fast enough rate to follow the aggregation process accurately. Despite these aforementioned challenges the Marsh laboratory has recently demonstrated the utility of ^19^F NMR to access direct, sensitive and real-time observation of amyloid fibril formation, enabling the elucidation of the underlying mechanics of peptide fibrillation.

**Figure 10:**
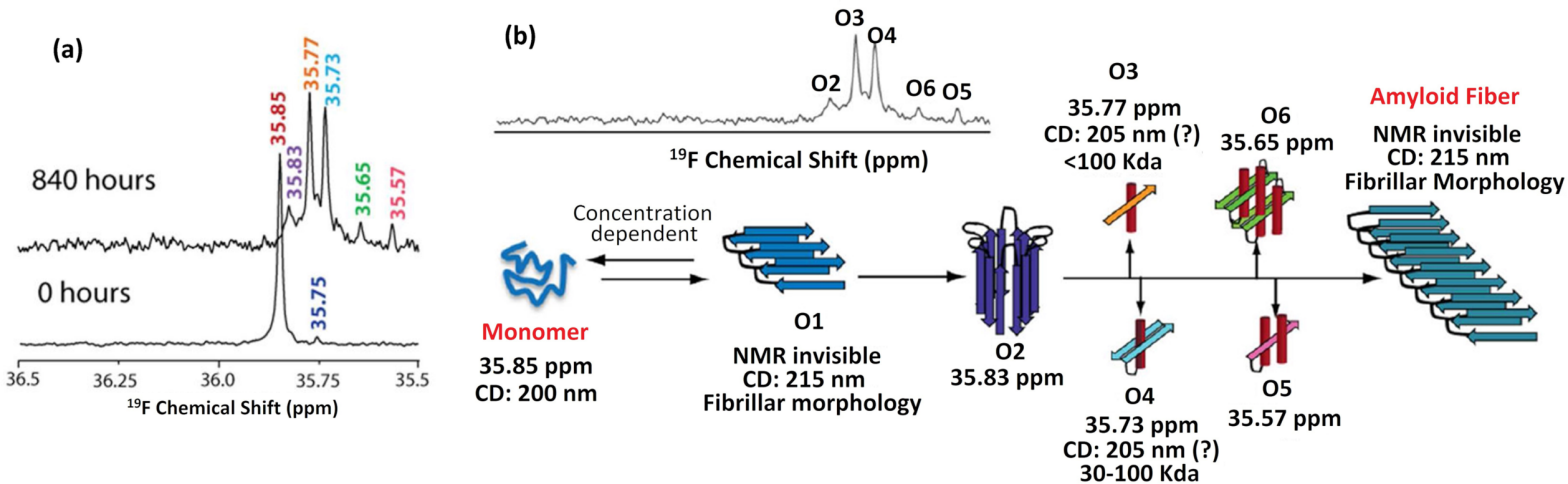
(a) Representative spectrum of a 182 μM sample of Aβ1-40-tfM35 at varying times indicating the major and minor peaks observed upon sample incubation. (b) Scheme describing the proposed aggregation pathway for Aβ1-40-tfM35. Adapted and reprinted with permission from [62]. © 2013 American Chemical Society.

To study the aggregation mechanism of the model Aβ-peptide, a well-known amyloidogenic peptide related to Alzheimer’s disease, Marsh and collaborators synthesized a ^19^F-labelled Aβ1−40 peptide version in which methionine at position 35 was replaced by tfmMet (Aβ1−40-tfmMet35) [9,62]. The aggregation process was then monitored by ^19^F NMR over a period of several weeks [62]. Upon analysis, formation of at least six spectroscopically distinguishable intermediates could be detected during fiber formation (Figure 10). Each independent intermediate was characterized on the basis of its ^19^F NMR chemical shift and the kinetics by which each species formed or decayed were evaluated in real-time. By combining the ^19^F NMR data with that provided by complementary analytical techniques, mass spectrometry (electrospray ionization and ion mobility), CD spectroscopy and atomic force microscopy, the authors were able to obtain detailed information about the size and secondary structure associated with some of the soluble intermediates. These included: large β-sheet oligomers formed immediately after solubilization (oligomer O1); a small oligomer that forms transiently during the early stages of the lag phase (oligomer O2) and 4 spectroscopically distinct forms of oligomers that appear during the later stages of aggregation and apparently coexist with the proto-fibrillar species (oligomers O3–O6, Figure 10).

It is worth noting that the ability to follow the formation and decay of multiple intermediate species using a single ^19^F NMR 1D experiment provides a straightforward route for the comparative analysis of different complex aggregation behaviours. For example, another study by the Marsh group carried out using amyloidogenic tfmPhe-labelled islet amyloid polypeptide hormone (tfmF-IAPP), showed that fibril formation can indeed proceed through well differentiated and distinguishable alternative pathways [69]. In this case, the consumption of ^19^F-labelled monomeric tfmF-IAPP was monitored in real-time by ^19^F NMR but significantly no new ^19^F resonances were produced during the time course of aggregation. This suggests that, unlike what is observed in the case of amyloidogenic Aβ-peptide, no soluble intermediates accumulate in the aggregation pathway of IAPP.

Conformational polymorphism is also particularly important for the infectious amyloid particles known as prions [70] and this area has been recently investigated using a range of ^19^F NMR approaches [63]. By selectively introducing 3-FPhe labels into the human prion protein segment PrP(90−231)_β_ via induced auxotrophy [71], Prosser and co-workers have been able to characterize and quantify the relative populations of monomeric, octameric, and higher oligomer species that populate the pathway from the native PrP state to fibril formation. The equilibrium constants between states were also analysed as a function of both temperature and pressure, allowing to determine the enthalpic and entropic contributions to their transitions. In addition, ^19^F NMR saturation transfer experiments allowed a convenient estimate of the kinetic rates at which the various species interconvert. This study by Prosser et al. nicely illustrates how quantitative and detailed thermodynamic and kinetic information for complex systems can be obtained by ^19^F NMR.

Another rapidly expanding and particularly challenging area of research where ^19^F NMR has found new applications is in the field of intrinsically disordered proteins (IDPs). IDPs play a key role in cell signalling and regulation processes and represent a distinct class of proteins that exhibit no stable 3D structure [72]. It is broadly understood that, unlike folded proteins, IDPs exist as an average combination of interconverting conformers. However, when bound to a substrate, IDPs may acquire a defined secondary structure [73]. The innate flexibility of IDPs imposes tremendous technical challenges to standard NMR analysis, as pronounced conformational averaging gives rise to narrow signal dispersion and low signal-to-noise ratios. Indeed, unless more sophisticated NMR techniques are employed, the combination of these two effects prevents the characterization of low-populated and short-lived states that might be critical for the peptide biological function [74].

**Figure 11:**
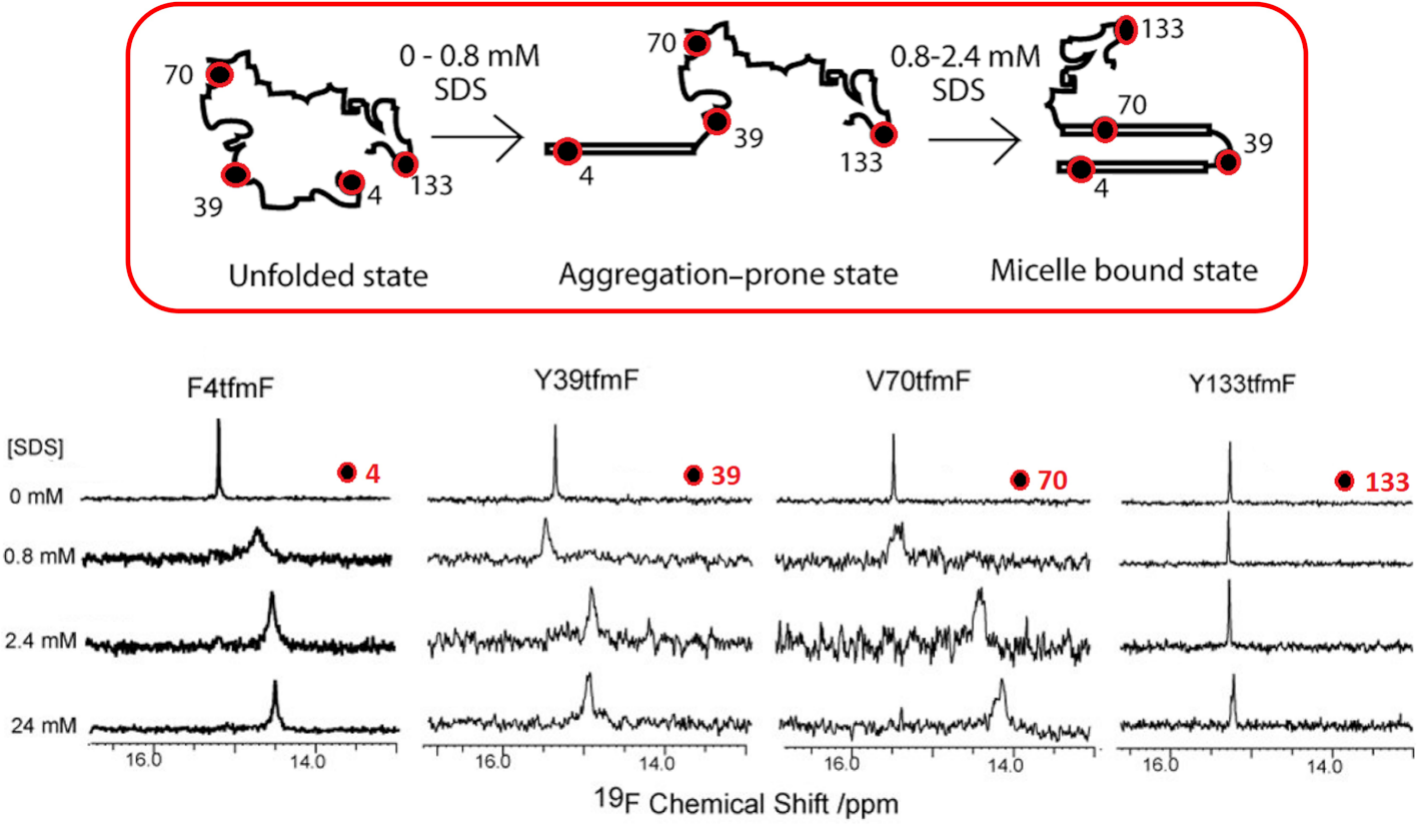
Illustration of the conformational switch induced by SDS in 4-tfmF-labelled α-Syn. Also shown are the ^19^F NMR spectra of the corresponding 4-tfmF-labelled peptides at positions 4, 39, 70 and 133 in varying concentrations of SDS. Figure 11 is reproduced from [60]. Copyright © 2010 WILEY‐VCH Verlag GmbH & Co. KGaA, Weinheim. Used with permission from Gui‐Fang Wang et al., “Probing the Micelle‐Bound Aggregation‐Prone State of α‐Synuclein with ^19^F NMR Spectroscopy”, ChemBioChem, John Wiley and Sons.

The Pielak group carried out an early study on the conformational dynamics of the model IDP, α-synuclein (αSyn) using ^19^F NMR. In this work ^19^F NMR was employed to study several properties of a specifically 3-FTyr-labelled α-synuclein analogue, including its native conformation and the conformational changes induced by urea, spermine and sodium dodecyl sulfate (SDS) [75]. Subsequently, α-synuclein interaction with SDS micelles and model membranes have also been investigated in detail [59,76], as well as the kinetics of α-Syn oligomerization and fibril formation both in vitro [60,75] and in vivo [77–78]. In these more recent studies, incorporation of 4-tfmF residues using amber-suppressing codons at various positions was shown to be more advantageous as a ^19^F reporting strategy, owing to the higher sensitivity and improved NMR relaxation properties of the CF_3_- group. The results from these studies, summarized in Figure 11, show that while α-Syn remains disordered in solution, it acquires partial helical secondary structure in the presence of SDS and membrane-like environments. Specifically, the N-terminal region of the peptide, which includes the first 4-tfmF-labelled amino acid, was shown to be involved in the most important membrane-binding interactions and conformational changes observed, allowing the protein to adopt a metastable aggregation-prone state that is apt to stabilize further intermolecular interactions and progress to the fibril state. The C-terminus non-amyloid component region, as monitored at position 133, was in comparison found to keep largely dynamically disordered in all species formed. Moreover, results from these studies revealed that the fibril-forming path of α-Syn might occur without accumulation of soluble low molecular weight intermediates, as no new ^19^F NMR signals were observed during the time-course of the aggregation process.

**Figure 12:**
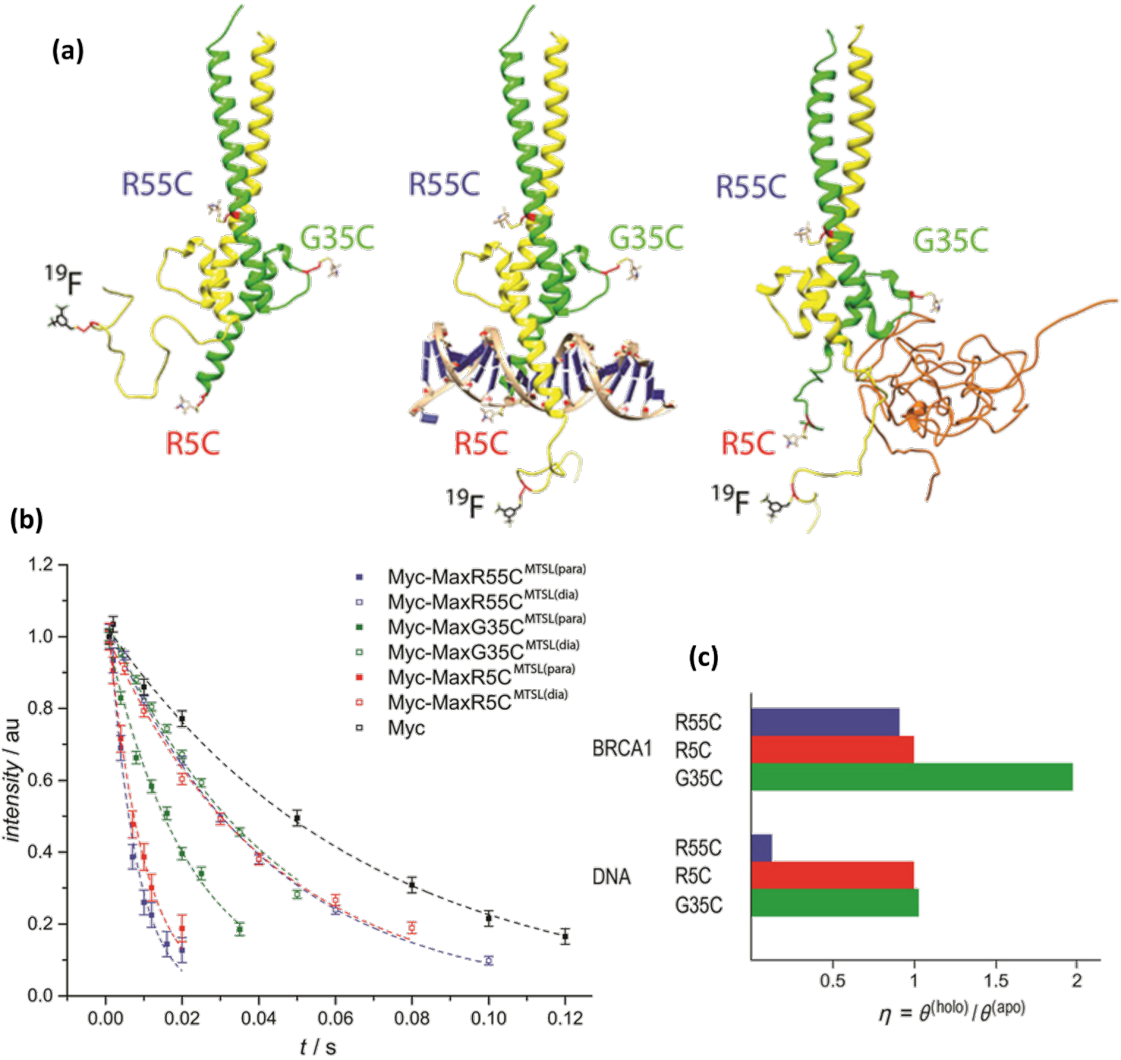
(a) Structural models of the Myc‐Max (left), Myc‐Max‐DNA (middle) and Myc‐Max‐BRCA1 complexes (right). Myc is shown in yellow, Max in green, BRCA1 in orange. (b) ^19^F T_2_ relaxation curves of tagged ^CF3^Myc and tagged ^CF3^Myc in complex with three Max mutants. (c) Relative PRE rates of different ^CF3^Myc‐Max‐BRCA1 and ^CF3^Myc‐Max‐DNA complexes. η quantifies the relative PRE effects in the Myc complex and monomeric Myc, which was normalized to diamagnetic relaxation rates. Figure 12 is reproduced from [79]. © 2019 Wiley‐VCH Verlag GmbH & Co. KGaA, Weinheim. Used with permission from Máté Somlyay et al., “^19^F NMR Spectroscopy Tagging and Paramagnetic Relaxation Enhancement‐Based Conformation Analysis of Intrinsically Disordered Protein Complexes”, ChemBioChem, John Wiley and Sons.

^19^F NMR spectroscopy has also now been employed to analyse the conformational dynamics of IDPs upon tertiary and quaternary complex formation [79]. The Myc-Max heterodimer is a well-known oncogenic transcription factor complex able to bind to enhancer box (E-box) regions (5’-CACGTG-3’) of DNA with low-nanomolar affinity, what triggers its biological function as a transcriptional regulator [80]. Previous studies have shown that in the monomeric state the two helical domains of Myc display unstable structural features, similar to those of small IDPs, existing rather as a highly flexible pair of transient α-helices [81]. Based on this, Konrat and co-workers explored a combination of ^19^F tagging and PRE NMR spectroscopy to probe and trace the conformational changes experienced by disordered Myc upon Myc‐Max heterodimerization. Going beyond, they also employed ^19^F NMR to interrogate the changes induced in the Myc-Max heterodimer structure upon full quaternary complex formation in the presence of DNA, and another intrinsically disordered binding partner, breast cancer antigen 1 (BRCA1) (Figure 12). In this work the strategy employed involved the introduction of a perfluorinated [^19^F]3,5‐bis(trifluoromethyl)benzyl-based tag into the single cysteine residue of Myc. This modification boosted the overall spectral sensitivity even when minimal protein concentrations were employed. The structural dynamics of the Myc-Max dimer formation were then evaluated by using intermolecular PREs between ^19^F‐Myc and three differently paramagnetic spin labelled MTSL ([(1-oxyl-2,2,5,5-tetramethyl-Δ3-pyrroline-3-methyl) methanethiosulfonate]) tagged Max mutants, and novel insights revealed regarding the differential structural dynamics of Myc‐Max bound to DNA and the tumour suppressor BRCA1. Given its ease of implementation, future applications of this strategy to new structural biology targets and inhibitor screening can be expected.

**Figure 13:**
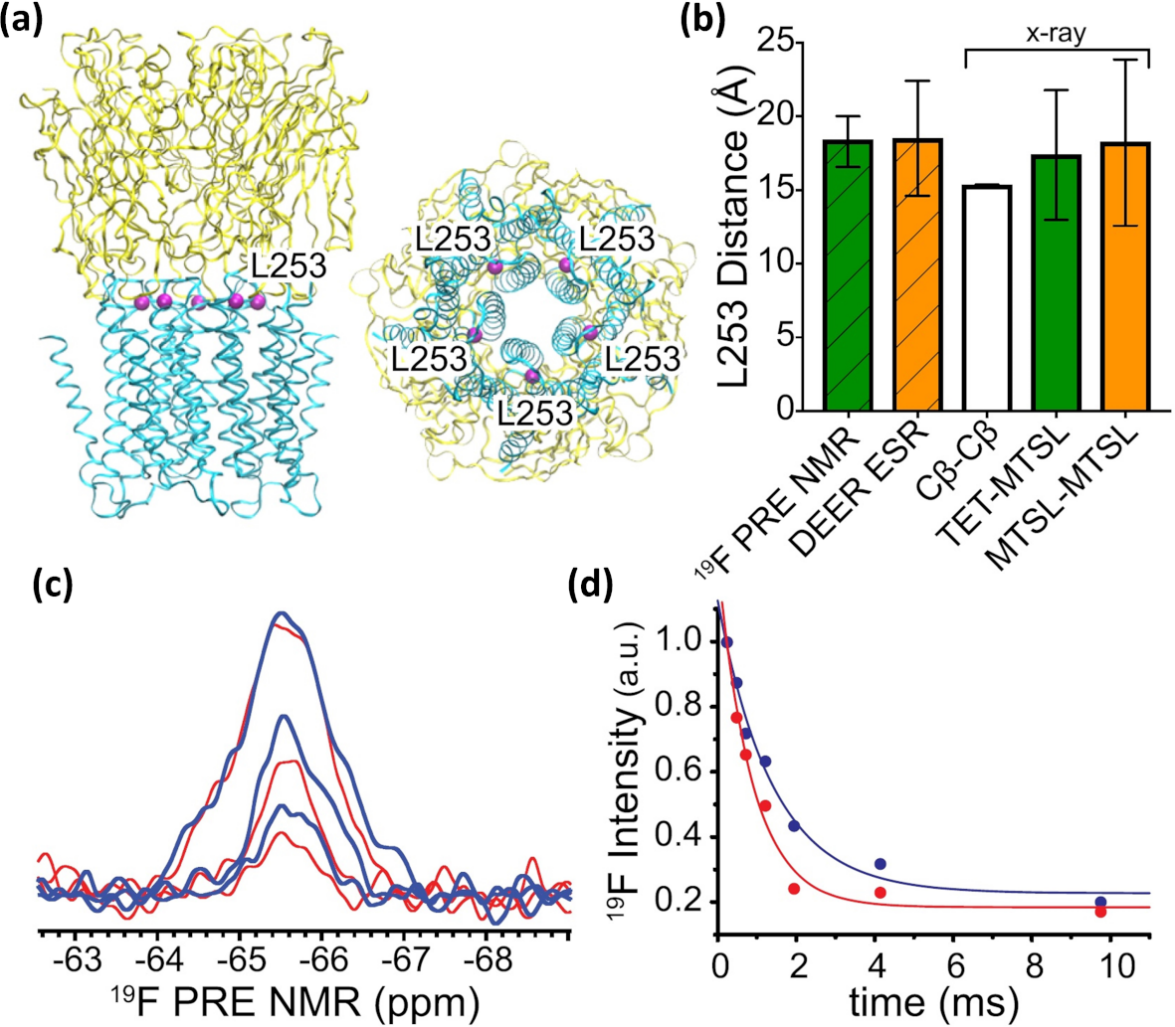
(a) Side (left) and bottom (right) views of the pentameric apo ELIC X-ray structure (PDB ID: 3RQU) showing the five equivalent L253 residues (purple) at the interface of the extracellular domain (yellow) and the transmembrane domain (cyan) present. (b) Distances obtained from ^19^F PRE NMR and DEER ESR experiments are compared to distances between L253 Cβ atoms (C_β_–C^β^) in adjacent subunits of the ELIC structure. (c) Representative ^19^F PRE NMR spectra of ELIC L253C-labelled with TFET and MTSL collected under paramagnetic (red) and diamagnetic (blue) conditions different with relaxation delays. (d) Single exponential decay functions, resulting in transverse relaxation rates of *R*_2,para_ = 1153 ± 194 Hz and *R*_2,dia_ = 714 ± 123 Hz were used to derive a distance of 18.4 ± 1.7 Å between residues 253 in the adjacent ELIC subunits. Reprinted with permission from [88]. © 2019 American Chemical Society.

In the field of ^19^F NMR protein conformational studies another area that has seen increasing attention is that of membrane proteins (MPs). Despite their physiological importance, analysis of the structural biology of MPs is significantly limited due to difficulties associated with protein expression, purification, stability, solubility and structural heterogeneity [82–84]. In particular, the application of solution NMR techniques to the study of MPs has proven exceptionally challenging due to the slower tumbling rates that MPs experience within membrane mimetics environments, which results in rapidly decaying signals and generally broad linewidths [85–86]. However, technical improvements, such as cryogenically-cooled probe-heads, along with the development of new high sensitivity ^19^F NMR probes and novel strategies to produce recombinant proteins with optimal isotope labelling have greatly opened up this field of study [87]. For example, by using highly sensitive 2,2,2-trifluoroethanethiol (TFET) ^19^F probes, Bondarenko et al. have developed a novel TFET/MTSL ((1-oxyl-2,2,5,5-tetramethylpyrroline-3-methyl)methanethiosulfonate) orthogonal labelling scheme that enables the accurate determination of inter-subunit distances in pentameric ligand-gated ion channels (pLGICs) by means of solution ^19^F PRE NMR experiments in micelles (Figure 13) [88]. To ensure a uniform ^19^F PRE signal from the adjacent paramagnetic labels, the ^19^F NMR TFET probe was tagged to a selected cysteine residue in the channel protein, L253C, and the paramagnetic probe, MTSL, was then used to label the rest of available equivalent cysteine sites in a molar ratio of 1 TFET:4 MTSL. The experimental distances, that were calculated on the basis of the corresponding ^19^F NMR signal decay profiles, were in good agreement to those predicted for modelled MTSL-TFET pairs in adjacent subunits in the X-ray structure, showing only small discrepancies. Overall, the results of this work clearly demonstrated the value of solution ^19^F NMR for quaternary structure determination and as an alternative approach for generating distance restraints for ion channels and other protein complexes that would be difficult to be defined by using other analytical tools.

#### DNA and RNA secondary and tertiary structure

^19^F NMR spectroscopy also represents a useful analytical approach to study the structure, function and molecular dynamics of nucleic acids [89]. Following early advances by Micura and co-workers in the late 2000s that investigated the suitability of ^19^F NMR for the conformational analysis of single and double helix RNA strands [90–93] ^19^F NMR has found a particularly rich niche of applications in the conformational analysis of higher-ordered DNA and RNA G-quadruplex structures. G-quadruplexes are four-stranded nucleic acid secondary structures formed in specific guanine-rich sequences showing, in general, highly polymorphic structures and various folding topologies. These structures have been suggested to play an important role in key biological processes such as gene expression and regulation [94–95], telomere length maintenance [96–98], transcription and DNA replication [99–100]. Investigation of the specific G-quadruplex structures associated with these biological events is therefore essential to understand their functions. However, because the formation of a stabilized G-quadruplex causes an overall reduction in the tumbling rates of the molecule, and thus a great decay of NMR signal sensitivity, analysis of these supramolecular structures by common NMR techniques has proved challenging.

**Figure 14:**
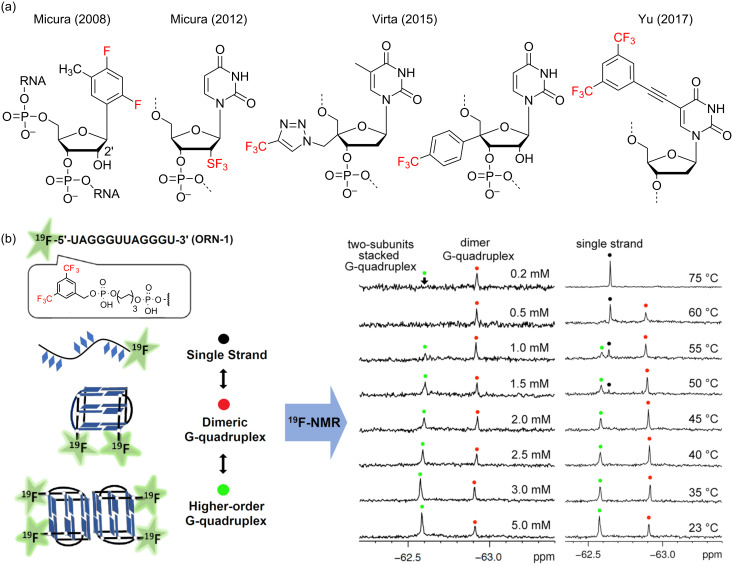
(a) General structure of a selection of recently developed ^19^F-labelled nucleotides for their use as ^19^F NMR reporters. (b) Concept for the detection different RNA structures by using ^19^F labels and its application to the study of telomer RNA structure at different concentrations and temperatures. Red and green spots indicate formation of dimer and two-subunits stacked G-quadruplex. The peaks of single strand RNA are marked with black spots. Temperatures are indicated on the right. Figure 14 is adapted from [102].

To overcome the aforementioned issues, Virta and co-workers have explored the application of trifluoromethyl analogues of guanosine, cytidine and uridine based in 2^’^-*O*-[(4-trifluoromethyltriazol-1-yl)methyl] reporter groups as ^19^F NMR probes for the detection of RNA secondary structures (Figure 14). As shown by Granqvist et al. [101], the ^19^F NMR signals observed when employing these probes to evaluate the thermal denaturation of a range of RNA hairpins were indeed found to be sensitive enough to allow the monitoring of their secondary structural changes with relatively wide shift dispersion. It also enabled to characterize by ^19^F NMR spectroscopic methods an RNA triple helix for the first time.

Following on from this a new range of even more sensitive ^19^F NMR probes, based in the use of 3,5-bis(trifluoromethyl)benzene moieties have recently been reported (Figure 14a) [103]. Here, the presence of six equivalent ^19^F atoms within the probe results key in providing a much more superior spectral enhancement, proving successful in enabling to monitor the conformational changes experienced within the DNA/RNA strains upon G-quadruplex formation both in vitro and in vivo. In addition, these novel probes have the extra advantage that can be easily incorporated either as internally fluorinated nucleobases or as external ^19^F-labelled terminal tags in longer oligonucleotides (Figure 14b). As a proof of concept, Bao et al. demonstrated the utility of these tags for the direct observation and quantitative thermodynamic characterization of dimeric and two-subunits stacked telomeric RNA and DNA G-quadruplexes within in living cells [99,102,104]. Moreover, by using these new reporters, ^19^F NMR analysis has also been applied as an efficient strategy to probe and characterize the binding interactions of fluoro-labelled RNA- and DNA-based G‐quadruplex complexes with different ligand molecules. This was exemplified by the Xu group who investigated the interaction of a RNA G‐quadruplex and the telomeric protein TRF2 [103] and the interaction of the DNA thrombin binding aptamer (TBA) G-quadruplex with thrombin [100]. Overall, these examples demonstrate that ^19^F NMR offers a suitable and non-perturbing approach by which to access detailed structural information of complex DNA and RNA folding topologies and sophisticated supramolecular assemblies.

### Metabolism studies

#### Biotransformation of fluorinated xenobiotics

**Figure 15:**
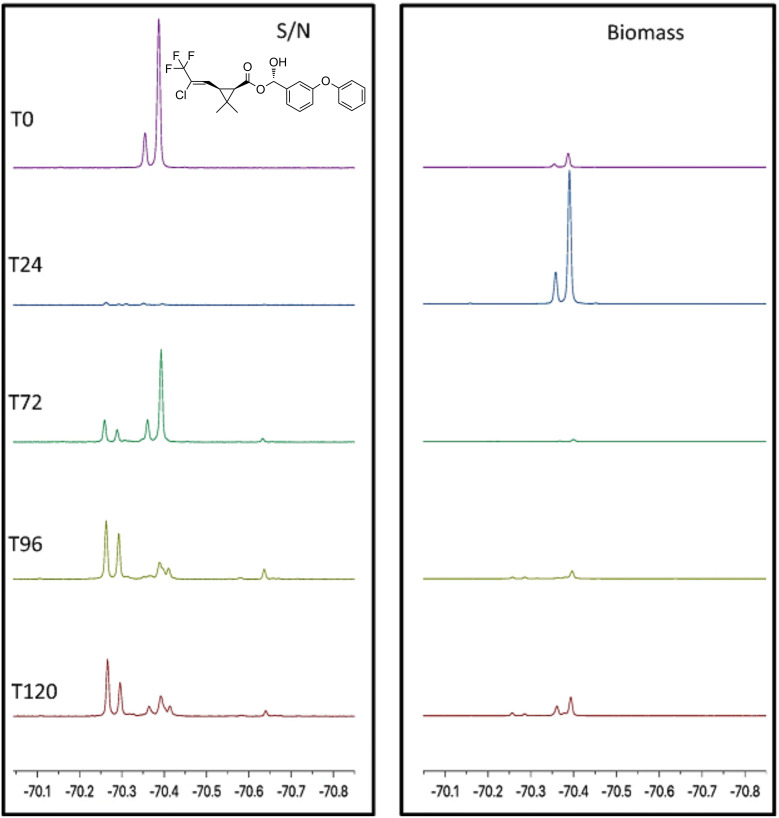
Monitoring biotransformation of the fluorinated pesticide cyhalothrin by the fungus *C. elegans*. The spectra are of supernatant (S/N) and biomass from cultures incubated with the pesticide at different time points. Figure 15 was reprinted from [106].

Fluorine is present in a large number of anthropogenic compounds, in particular pharmaceuticals, agrochemicals and anti-stain/anti-stick compounds (per- and poly-fluorinated alkyl substances, PFAS). When these compounds come into contact with microorganisms, there is a high likelihood that they will be biotransformed to some degree. In our previous review [4], we highlighted ^19^F NMR’s usefulness in following the biodegradation of compounds such as fluorophenols and fluorobenzoates. The technique has since been applied to monitor the biotransformation/biodegradation of fluorinated drugs such as flurbiprofen [105], and the pesticide cyhalothrin [106] by the fungus *Cunninghamella elegans*. In the former, ^19^F NMR demonstrated the appearance of phase 1 (oxidative) and phase 2 (conjugative) metabolites, and in the latter, it was possible to monitor the migration of the pesticide into the biomass in the first 24 h after its introduction before being biotransformed to new trifluoromethyl-containing metabolites (Figure 15). ^19^F NMR was also employed to determine the degree of biotransformation of drug-like fluorophenylpyridine carboxylic acids in the same fungus [107]. However, aside from these few examples, the technique is not widely used by those investigating the biodegradation of fluorinated xenobiotics, with researchers more commonly relying on liquid or gas chromatography–mass spectrometry methods. Objectively, these techniques are much more sensitive than ^19^F NMR, provide more structural information and are probably more accessible; however, it is possible that important biotransformation products may not to be detected using these techniques. For example, the fluorometabolites detected upon fungal degradation of cyhalothrin were not observed by GC–MS of culture supernatants.

**Figure 16:**
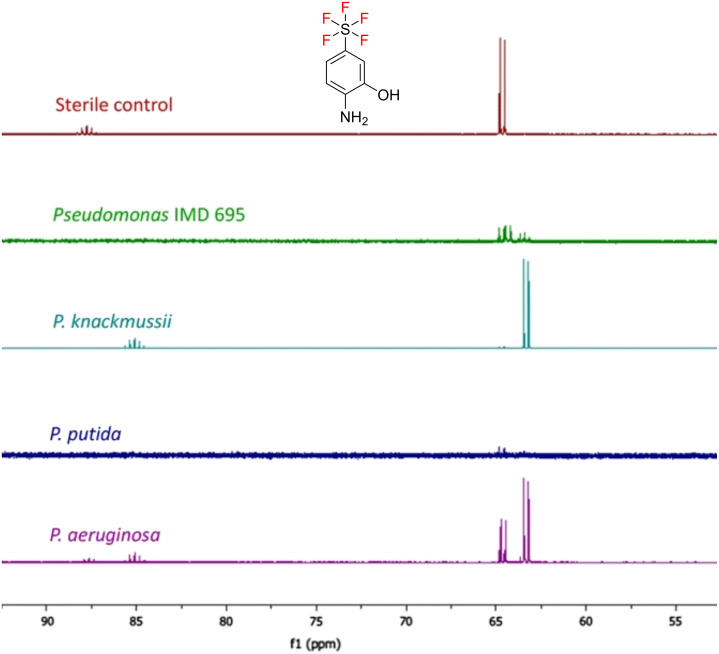
Following the biodegradation of emerging fluorinated pollutants by ^19^F NMR. The spectra are from culture supernatants of different bacteria incubated with SF_5_-aminophenol. Figure 16 was reprinted from [110].

When the nature of the degradation products is unknown, ^19^F NMR is particularly useful, since even minor changes to a substrate’s structure can lead to a chemical shift change. Notably, compounds with new fluorinated groups, for which there is no precedent in the literature, pose challenges for analysis of biotransformation products. For instance, the pentafluorosulfanyl (-SF_5_) group is proposed as a replacement for trifluoromethyl (-CF_3_) and has been incorporated into numerous biologically active compounds already [108–109]. Intuitively, investigating the biodegradation of these compounds in the absence of any reference compounds is complicated. Saccomanno et al. [110] investigated the bacterial degradation of pentafluorosulfanyl (SF_5_)-substituted aminophenols and via ^19^F NMR analysis of culture extracts was able to determine the production of new fluorinated metabolites (Figure 16). Subsequent analysis by GC–MS was required to further characterize the products, but only one could be detected (SF_5_-catechol) despite the ^19^F NMR analysis showing the presence of multiple fluorometabolites.

#### Detection and biosynthesis of natural organofluorine compounds

As naturally-occurring organofluorine compounds are so rare, it is possible to easily detect them in a crude, complex mixture, such as a culture medium, using ^19^F NMR. The earliest example of the technique being applied in this was in the discovery of fluoroacetate and 4-fluorothreonine in cultures of the bacterium *Streptomyces cattleya* [111]. ^19^F NMR became a cornerstone for investigating the biosynthesis of these fluorometabolites, and was instrumental in the discovery of the very first fluorinase, which catalyses the production of 5’-fluoro-5’-deoxyadenosine (5-FDA) from fluoride ion and *S*-adenosylmethionine (SAM) [1].

**Figure 17:**
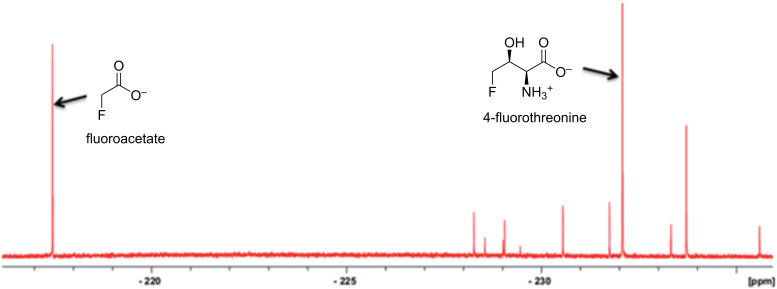
Discovery of new fluorinated natural products by ^19^F NMR. The spectrum is of the culture supernatant of *Streptomyces* sp. MA37, which shows new fluorometabolites in addition to the previously identified fluoroacetate and 4-fluorothreonine. Figure 17 was reprinted from [113].

While cumbersome as a tool for screening for new fluorometabolites in multiple strains, ^19^F NMR continues to be successfully applied to the discovery of such compounds, typically after genome sequencing and scanning for genes with homology to those initially discovered in *S. cattleya*. For example, Huang et al. [112] analysed the genome sequence of the bacterium *S. xinghaiensis* NRRL-B24674, which revealed the presence of a fluorinase gene, suggesting that the microorganism could produce fluorinated compounds from fluoride ion. This was confirmed by ^19^F NMR of culture supernatant after the bacterium was grown in medium containing 2 mM fluoride ion. Furthermore, because of the large shift changes due to minor structural differences, the observation of new fluorinated compounds in crude culture supernatants is possible. ^19^F NMR analysis of cultures of the bacterium *Streptomyces* sp. MA37, which was isolated from a Ghanian soil sample, revealed several new resonances in addition to those known for fluoroacetate and 4-fluorothreonine (Figure 17) [113]. (2*R*,3*S*,4*S*)-5-Fluoro-2,3,4-trihydroxypentanoic acid and 5-fluoro-5-deoxy-ᴅ-ribulose were confirmed as two of the new metabolites [114]. Most recently ^19^F NMR was the key analytical technique to assess in vivo production of 5’-FDA in an engineered *E. coli*, which, in addition to expressing the fluorinase, had its fluoride efflux protein, CrcB, deleted and a gene coding for a SAM transport protein heterologously expressed, to ensure an adequate supply of substrates [115].

**Figure 18:**
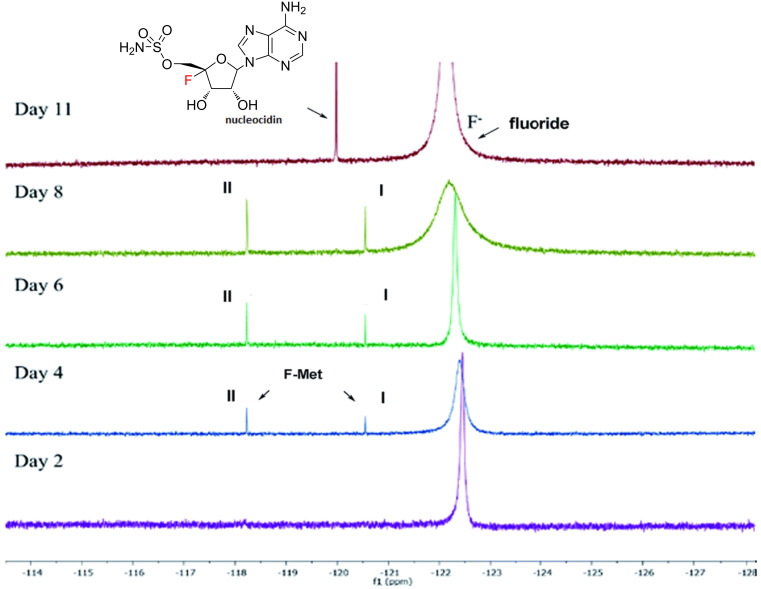
Application of ^19^F NMR to investigate the biosynthesis of nucleocidin. The spectra are from culture supernatants of *S. calvus* recorded at different times during growth, showing the production of two glycosylated metabolites (I and II) that precede nucleocidin appearance in the culture. Figure 18 was reprinted from [119].

Nucleocidin was famously isolated from *S. calvus* in the 1950s as it had significant anti-trypanosomal properties [116]; however, the presence of fluorine in its structure was not discovered until a decade later [117], by which time the original strains had lost their biosynthetic capability, frustrating attempts to investigate its biosynthesis. It took over 50 years to determine the reason for the loss of nucleocidin production, which was due to the presence of a point mutation in the bldA gene [118] that codes for a rare Leu-tRNA (UUA). Upon complementation with a functional gene, *S. calvus* produced nucleocidin once again, which was detected by ^19^F NMR. Subsequent biosynthetic investigations, again relying on ^19^F NMR, have revealed that the elaboration of this unusual fluorometabolite involves the production of glucosylated precursors [119], which are detectable in the culture supernatant (Figure 18). These are possibly inactive forms of nucleocidin, generated in the first instance so that the producing organism is protected from its deleterious effects, and can be activated outside the cell by glucosidases.

#### Fluorinated natural products via precursor-directed biosynthesis

**Figure 19:**
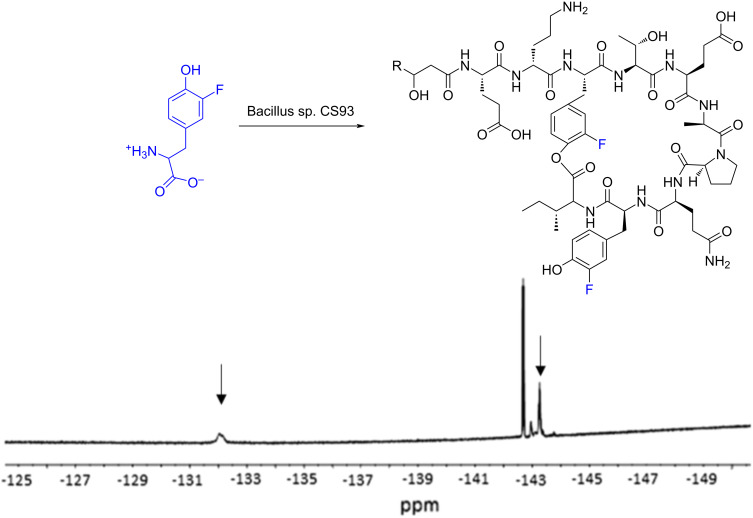
Detection of new fluorofengycins (indicated by arrows) in culture supernatants of *Bacillus* sp. CS93 incubated with 3-fluoro-ʟ-tyrosine with ^19^F NMR. Figure 19 was reprinted from [121].

The modification of known antibiotics and other natural products is an important tactic in the battle against antimicrobial resistance. In addition to fluorinated metabolites that are produced de novo from fluoride ions, it is possible to modify other secondary metabolites to incorporate fluorine by including fluorinated precursors in the culture medium. ^19^F NMR has been applied to the detection of the new compounds in the complex supernatants of organisms producing non-ribosomal peptides and polyketides. It has been especially useful for monitoring the incorporation of fluorinated amino acids into lipopeptides produced by *Bacillus* sp. CS93. This bacterial strain was originally isolated from the Mayan fermented food Pozol and was found to produce the dipeptide antibiotic bacilysin and the lipopeptides iturin A, surfactin and fengycin [120]. Precursor-directed biosynthesis experiments with various fluorinated amino acids were conducted and it was observed that in iturin A and fengycin, 3-fluoro-ʟ-tyrosine could replace tyrosine in the peptide chain [121–123]. ^19^F NMR analysis of culture supernatants revealed that when this fluoro-amino acid was incubated with CS93, three different fluorofengycin species were biosynthesised (Figure 19), with fluorotyrosine replacing either or both tyrosine residues in the peptide ring.

Although not strictly precursor-directed biosynthesis, Piasecki and Keatinge-Clay [124] employed ^19^F NMR to monitor biocatalytic reactions of trifluorinated surrogate substrates of different polyketide synthase activities in cell lysates of recombinant *E. coli*. Substrate and lysate were incubated in an NMR tube to enable continuous monitoring of reactions catalysed by erythromycin thioesterase (trifluoropropionyl-SNAC substrate) and various ketoreductases (3-oxo-5,5,5- and 2-methyl-3-oxo- trifluoromethylpentanoyl-SNAC substrates). ^19^F NMR successfully resolved the diastereomers produced by the different ketoreductases assayed.

#### Measuring gene expression

**Figure 20:**
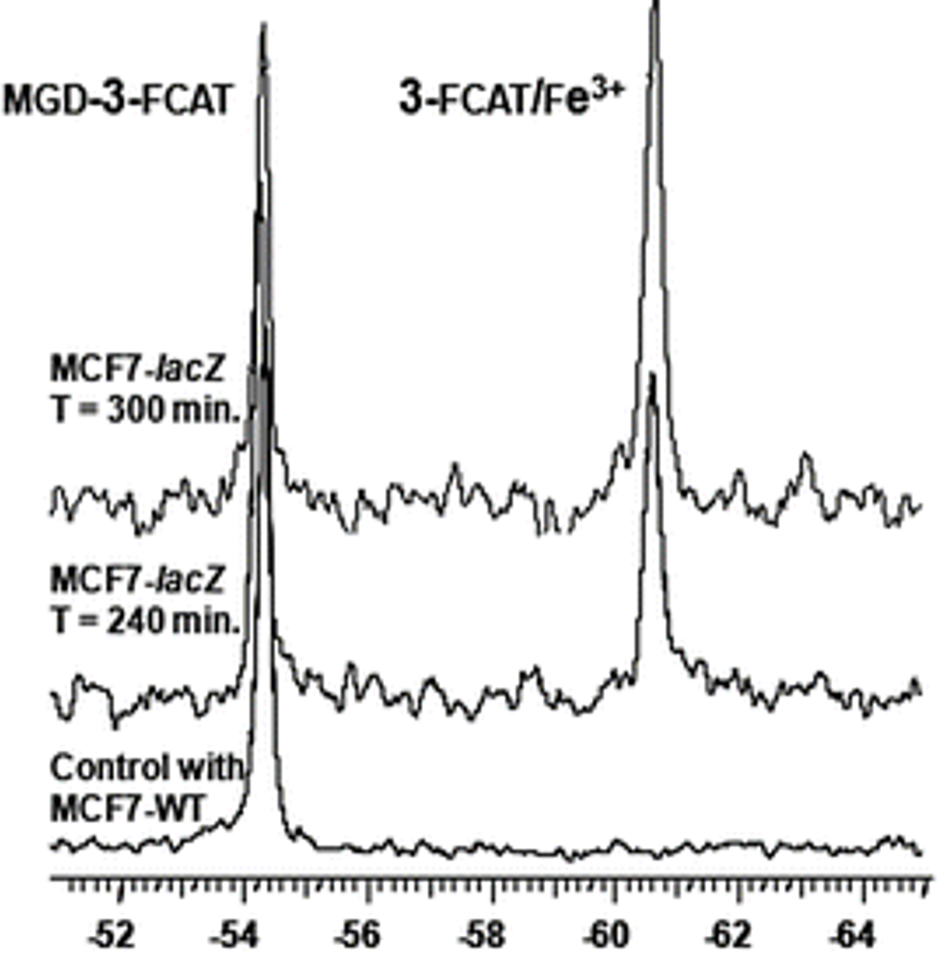
Measurement of β-galactosidase activity in MCF7 cancer cells expressing lacZ using ^19^F NMR. The deglycosylated FCAT probe binds the Fe^3+^ ions present resulting in a complex that gives a new resonance in the spectrum. Figure 20 was reprinted from [125].

The *lacZ* gene, which is part of the lac operon in bacteria such as *E. coli*, codes for β-galactosidase and is used extensively as a reporter of gene expression. The measurement of β-galactosidase activity is possible using a range of techniques, including ^19^F NMR. Yu et al. [125] developed a bimodal fluorinated probe, 1-*O*-(β-ᴅ-galactopyranosyl)-3-fluorocatechol, to measure the enzyme’s activity in transfected cancer cells. The principle relies on the excess Fe^3+^ ions characteristically present in tumour cells, which are scavenged by the catechol after it is released from the sugar via the action of β-galactosidase. The resulting complex has a different chemical shift to the original compound in the ^19^F NMR spectrum (Figure 20). The change could also be measured by ^1^H MRI (magnetic resonance imaging) allowing improved precision and reliability of the assay.

^19^F MRI can monitor gene expression in living cells by utilising cell-surface displayed β-lactamase (β-lac) and a specifically designed ^19^F MRI probe [126]. The probe comprised a trifluoromethoxylated β-lactamase substrate, cephalosporin, attached to a Gd^3+^ complex, but it cannot cross the cell membrane. To avoid the need for membrane permeabilization, β-lac was fused to an extracellular region of epidermal growth factor receptor (EGFR). The non-permeable probe can bind the reporter protein on the cell surface, allowing measurement of intracellular gene expression. Hydrolysis of the probe by β-lac released the Gd^3+^ complex causing the T_2_ of the ^19^F-containing group to increase owing to a loss in the intramolecular paramagnetic relaxation enhancement (PRE). The T_2_ values can affect the ^19^F MRI signal intensity, which can be visually identified and a reduction in the thickness of peaks in the ^19^F NMR spectra can also be observed. Therefore, enzymatic degradation of the Gd-FC-lac probe would lead to the improvement of the ^19^F MRI signal, confirming gene expression in living cells.

### Physiological measurement

#### Detection of ions

**Figure 21:**
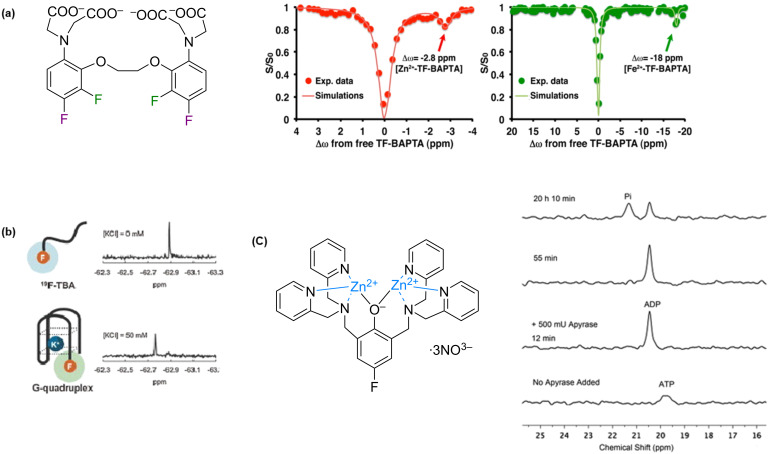
Detection of ions using ^19^F NMR. (a) Structure of TF-BAPTA and its ^19^F iCEST spectra in the presence of Zn^2+^ (red) or Fe^2+^ (green); (b) Detection of K^+^ upon complexation with trifluorinated thrombin aptamer forming a G-quadruplex.; (c) Structure of fluorinated Zn^2+^-dipicolylamine co-ordination complex and its application to the detection of phosphate released from ATP by apyrase. Figure 21a is reproduced with permission from [129], Copyright © 2014 American Chemical Society, https://pubs.acs.org/doi/10.1021/ja511313k. Further permissions related to the material excerpted should be directed to the ACS. Figure 21b is reprinted with permission from [130], © 2011 The Chemical Society of Japan. Figure 21c was reprinted from [131].

Metal ions play an important role in biological systems and deviations in their levels can be associated with the onset of disease. The real-time detection and tracking of metal ions in vivo are of great interest, which is not possible using optical dyes. One potential way to overcome this is to employ ^19^F-labelled chelators and sensors, whose chemical shifts are observed by ^19^F NMR upon detection or binding to the metal ion of interest. Early work by Smith et al. [127] employed symmetrically difluorinated 1,2-bis(*o*-aminophenoxy)ethane-*N*,*N*,*N*’,*N*’-tetraacetic acid (BAPTA) to determine intracellular Ca^2+^ concentration from the areas of the resonances of free and complexed forms of the chelating reagent. To improve sensitivity of the 5,5’-difluoro-BAPTA (5F-BAPTA) for ^19^F MRI Bar-Shir et al. [128] used ion chemical exchange saturation transfer (iCEST). This approach is an extension of CEST that is used in MRI where a dynamic exchange between radiofrequency (RF) labelled protons and bulk water yields enhanced contrast. In iCEST, RF labelling at the ^19^F frequency of Ca^2+^-5F-BAPTA and detection of the label transfer to the frequency of free 5F-BAPTA produced a 100-fold improvement in sensitivity. A tetra-fluorine (5,5’,6,6’-tetrafluoro-) BAPTA (TF-BAPTA) ion receptor was designed and successful in the detection of Zn^2+^ and Fe^2+^ due to slower exchange rates of the free and bound probe observed for both metal ions (Figure 21a) [129].

The conformational changes of biomolecules caused by metal ions has been exploited in the design of ^19^F NMR-based ion sensors. For example, in the presence of K^+^, the thrombin aptamer (5’-GGTTGGTGTGGTTGG-3’) undergoes an intramolecular conformational change to promote the formation of the G quadruplex. A K^+^ sensor was designed by introducing a 3,5-bis(trifluoromethyl)benzene moiety at the 5’ terminal of the aptamer (^19^F-TBA) [130]. When KCl was added, a new ^19^F NMR signal was observed suggesting the complexation of K^+^ and a conformational change had occurred (Figure 21b). The sensor displayed excellent sensitivity as no new ^19^F NMR signals and no chemical shift changes were observed upon addition of other metal ions such as Li^+^, Na^+^, Mg^2+^ and Ca^2+^.

Anions, in particular phosphate, are also important physiological markers, and ^19^F NMR can be employed for their detection. Gan et al. [131] reported the chemical shift changes upon phosphate anions binding to a Zn^2+^-dipicolylamine co-ordination complex and used it to follow ATP hydrolysis by apyrase (Figure 21c).

#### Detection of biological reactive oxygen species

Reactive oxygen species (ROS), such as hydrogen peroxide (H_2_O_2_), superoxide (O_2_^−^), and hydroxyl radical (•OH), are produced as part of mitochondrial oxidative metabolism and as a response to cellular invasion by cytokines, xenobiotics, and bacteria [132]. H_2_O_2_ is considered to be one of the most important ROS and play a role in homeostatic regulation as well as in healthy physiological signalling pathways such as cell proliferation, differentiation and migration. Fluorescent probes are typically used to detect H_2_O_2_ in living organisms and although highly sensitive, the excitation and emission light are unable to penetrate deep sites in the body.

Arylboronic acids are oxidised by H_2_O_2_, allowing for the design of H_2_O_2_-responsive sensors with these substrates as reactive moieties [133]. ^19^F-labelled phenylboronic acid was used as a substrate in the design on a ^19^F MRI probe to detect H_2_O_2_ [134]. The fluorinated phenylboronic acid and the H_2_O_2_ interact to form a corresponding phenol, resulting in a large ^19^F chemical shift change due to the large electron density change around the ^19^F nucleus.

**Figure 22:**
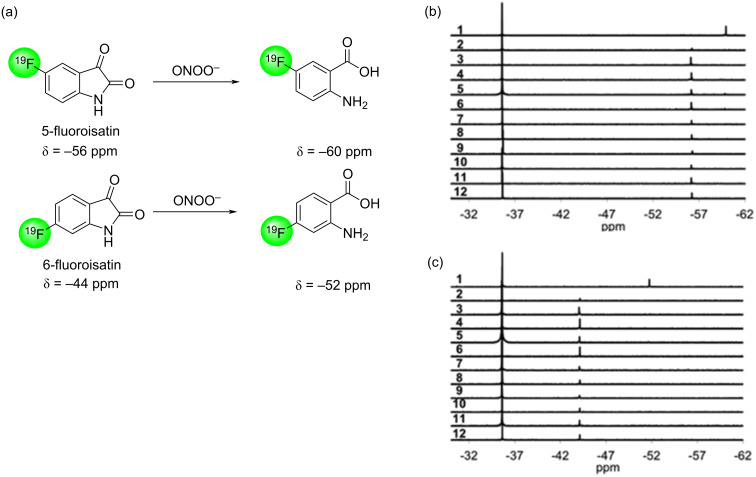
(a) The ONOO^−^-mediated decarbonylation of 5-fluoroisatin and 6-fluoroisatin. The selectivity of (b) 5-fluoroisatin and (c) 6-fluoroisatin to reactive oxygen species. 1: ONOO^−^, 2: OH, 3: GSH, 4: NO, 5: Na_2_N_2_O_3_, 6: KO_2_, 7: *t*-BuOOH, 8: GSNO, 9: NO_2_^−^, 10: ClO^−^, 11: KHSO_5_, 12: H_2_O_2_. Figure 22 was reprinted from [135].

Peroxynitrite (ONOO^−^) is a highly reactive nitrogen oxide species formed in vivo from the rapid interaction of O_2_^−^ and nitric oxide radicals. •NO and O_2_^−^ are endogenously formed to moderate cell signalling, whereas ONOO^−^ production is thought to be harmful with increased levels observed in cancer and age-related pathologies. Detection ONOO^−^ in biological samples is difficult owing to its short lifetime, strong competition from endogenous ROS scavengers and high background noises from other ROS. Two ^19^F magnetic resonance probes, 5-fluoroisatin and 6-fluoroisatin, were developed for the detection of ONOO^−^ based on oxidative carbonylation chemistry [135]. Both probes are highly selective for ONOO^−^, resulting in a chemical shift change in ^19^F NMR, which was not observed when the probes were incubated with other reactive sulphur, oxygen and nitrogen species (Figure 22).

## Conclusion

In the decade since our previous review, ^19^F NMR has continued to prove its versatility in several areas of chemical biology, but predominantly in the structural analysis of protein and nucleic acid, and their interactions with other biomolecules. Advances in the methods for the synthesis of fluorinated amino acids and nucleotides have been the driver for the new applications, coupled with improved sensitivity of the instrumentation. The recognition of the importance of structural biology in disease states is likely to amplify the application of ^19^F NMR in this field. Undoubtedly, the technique will continue to be employed in all of the areas highlighted in this review, and new applications identified since fluorine’s importance across a range of industries is undiminished.
